# Depletion of polyfunctional CD26^high^CD8^+^ T cells repertoire in chronic lymphocytic leukemia

**DOI:** 10.1186/s40164-023-00375-5

**Published:** 2023-01-27

**Authors:** Najmeh Bozorgmehr, Mark Hnatiuk, Anthea C. Peters, Shokrollah Elahi

**Affiliations:** 1 School of Dentistry, Division of Foundational Sciences, University of Alberta, Edmonton, AB T6G 2E1 Canada; 2 Depatment of Medicine Division of Hematology, University of Alberta, Edmonton, AB T6G 2E1 Canada; 3 Department of Oncology, Division of Medical Oncology, University of Alberta, Edmonton, AB T6G 2E1 Canada; 4 Li Ka Shing Institute of Virology, Faculty of Medicine and Dentistry, University of Alberta, Edmonton, AB T6G 2E1 Canada

**Keywords:** MAIT cells, Galectin-9, T cells exhaustion, Adoptive T cell therapy

## Abstract

**Background:**

CD8^+^ T cells play an essential role against tumors but the role of human CD8^+^CD26^+^ T cell subset against tumors, in particular, haematological cancers such as chronic lymphocytic leukemia (CLL) remains unknown. Although CD4^+^CD26^high^ T cells are considered for adoptive cancer immunotherapy, the role of CD8^+^CD26^+^ T cells is ill-defined. Therefore, further studies are required to better determine the role of CD8^+^CD26^+^ T cells in solid tumors and haematological cancers.

**Methods:**

We studied 55 CLL and 44 age-sex-matched healthy controls (HCs). The expression of CD26 on different T cell subsets (e.g. naïve, memory, effector, and etc.) was analyzed. Also, functional properties of CD8^+^CD26^+^ and CD8^+^CD26^−^ T cells were evaluated. Finally, the plasma cytokine/chemokine and Galectin-9 (Gal-9) levels were examined.

**Results:**

CD26 expression identifies three CD8^+^ T cell subsets with distinct immunological properties. While CD26^neg^CD8^+^ T cells are mainly transitional, effector memory and effectors, CD26^low^CD8^+^ T cells are mainly naïve, stem cell, and central memory but CD26^high^ T cells are differentiated to transitional and effector memory. CD26^+^CD8^+^ T cells are significantly reduced in CLL patients versus HCs. CD26^high^ cells are enriched with Mucosal Associated Invariant T (MAIT) cells co-expressing CD161TVα7.2 and IL-18Rα. Also, CD26^high^ cells have a rich chemokine receptor profile (e.g. CCR5 and CCR6), profound cytokine (TNF-α, IFN-γ, and IL-2), and cytolytic molecules (Granzyme B, K, and perforin) expression upon stimulation. CD26^high^ and CD26^low^ T cells exhibit significantly lower frequencies of CD160, 2B4, TIGIT, ICOS, CD39, and PD-1 but higher levels of CD27, CD28, and CD73 versus CD26^neg^ cells. To understand the mechanism linked to CD26^high^ depletion, we found that malignant B cells by shedding Galectin-9 (Gal-9) contribute to the elevation of plasma Gal-9 in CLL patients. In turn, Gal-9 and the inflammatory milieu (IL-18, IL-12, and IL-15) in CLL patients contribute to increased apoptosis of CD26^high^ T cells.

**Conclusions:**

Our results demonstrate that CD26^+^ T cells possess a natural polyfunctionality to traffic and exhibit effector functions and resist exhaustion. Therefore, they can be proposed for adoptive cancer immunotherapy. Finally, neutralizing and/or inhibiting Gal-9 may preserve CD26^high^CD8^+^ T cells in CLL.

**Supplementary Information:**

The online version contains supplementary material available at 10.1186/s40164-023-00375-5.

## Key messages

- CD26^high^CD8^+^ cells as potential adoptive immunotherapy candidate
- CD26^high^CD8^+^ T cells are enriched with MAIT cells
- Galectin-9 preferentially induces apoptosis in CD26^high^CD8^+^ T cells
- Malignant B cells are a source of Galectin-9

**Supplementary Information:**

The online version contains supplementary material available at 10.1186/s40164-023-00375-5.

## Background

CD26 also known as DPP4 (dipeptidyl peptidase 4) is a 110 kDa homodimer transmembrane glycoprotein with enzymatic activity [1]. It has extracellular, transmembrane, and intracytoplasmic domain [1]. The extracellular domain contains catalytic, cysteine-rich, and glycosylated regions. The catalytic region has serine protease activity that cleaves off amino-terminal dipeptides from many peptide hormones and chemokines that have proline or alanine at their N-terminus [2]. Also, CD26 stabilizes glucose levels by inactivating glucagon-peptide-1 (GLP) and gastric-inhibitory protein (GIP) [3]. The glycosylated and cysteine-rich regions of CD26 interact with different binding partners [4].

CD26 is widely expressed by various cells in different tissues including fibroblasts, endothelial, epithelial, mesothelial, and immune cells [5]. Among immune cells, T cells are the major CD26 expression cells [6]. Nevertheless, B cells [7], natural killer cells (NK) [8], dendritic cells (DCs), [9] and macrophages [10] express lower levels of CD26. It is worth mentioning that erythroid precursors/progenitors (CD71^+^ erythroid cells) also express substantial levels of CD26 [11].

The role of CD26 in the immune system particularly T cell development and differentiation has been widely studied. For example, CD26 is considered a thymus maturation marker for T cells since most single-positive CD4^+^ and CD8^+^ T cells express this glycoprotein [12]. Notably, deletion of CD26 in mice is associated with decreased frequency and functionality of CD4^+^ T cells, which subsequently impairs cytokine and immunoglobulin production in CD26 knock-out (KO) mice [13]. In line with these observations, CD26 (DPP4) inhibitors, as glycemic controllers have been associated with Th1, Th2, and Th17 cell suppression but regulatory T cells (Tregs) expansion in diabetic patients [14].

CD26 modulates T cell activation and proliferation via interaction with its binding partners such as Adenosine Deaminase (ADA), which is an essential enzyme in the adenosine pathway [15]. Upon binding to CD26, ADA converts adenosine to inosine with a wide range of anti-inflammatory effects [16, 17]. Moreover, the interaction of ADA with CD26 transduces a stimulatory signal to T cells [18]. For instance, Caveolin-1 on antigen -presenting cells upon interaction with CD26 via CARMA-1 enhances T cell activation [19].

CD26 is also involved in T cell trafficking by modulating chemokines and the tendency for binding to extracellular matrix molecules and endothelial cells [20]. The enzymatic activity of CD26 regulates diverse chemokines including RANTES (CCL5), Eotaxin, Stromal-derived Factor-$\alpha$ (SDF-1 $\alpha$/CXCL12), and macrophage-derived chemokine (MDC/CCL22) [21]. For example, CD26 increases CCR5-dependent but reduces CCR1-dependent migration of monocytes [22]. Additionally, the CD26 molecule has binding sites for extracellular matrix components such as fibronectin and collagen [23]. These capabilities support CD26^+^ T cell activity, homing and trans-endothelial migration [24].

It appears that CD26^high^CD4^+^ T cells are dominantly Th17 cells and exhibit effective anti-tumor immunity in different cancer models [25–27]. CD26^high^CD4^+^ T cells, due to their increased migration and persistence capacities, are desirable for T cell-based immunotherapies [26]. It is worth mentioning that CD26 expressing CD4^+^ T cells play a crucial role in the differentiation of B cells into plasma cells [6].

However, compared to CD4^+^ T cells, the role of CD26 in CD8^+^ T cell is not fully understood. Some studies have reported that CD26 provides a costimulatory signal and increases cytokine production in CD8^+^ T cells [28]. Given its costimulatory role, CD26 blockade attenuates organ transplantation [29, 30] and skin allograft rejections in animal models [31, 32]. Moreover, CD26 plays an essential role in the formation of memory CD8^+^ T cells in viral infections [33] but their frequency is reduced in HIV-infected individuals [34]. Nevertheless, the loss of CD26 expression in malignant T cells in cutaneous T cell lymphoma is reported [35]

Recently it has been shown that CD26 expression is a distinctive surrogate marker for Mucosal Associated Invariant T (MAIT) cells [36]. Human MAIT cells are unconventional innate-like T cells that are present in blood circulation and tissues [37]. These cells are defined by the expression of a semi-invariable T-cell receptor-$\alpha$ chain (TCR-$\alpha$) composed of TV $\alpha$ 7.2[38]. MAIT cells are restricted to MHC class I related Protein 1 (MR-1) which enables them a unique opportunity to recognize riboflavin (Vitamin B12) metabolites in microbial components [39]. In addition, MAIT cells exhibit a distinct phenotype evidenced by high levels of surface markers such as CD161, IL-18R $\alpha$, and CD26 [36, 40, 41]. However, to our knowledge, the frequency and functionality of CD26^+^CD8^+^ T cells in Chronic Lymphocytic Leukemia (CLL) have never been investigated.

CLL is a hematologic malignancy with clonal expansion of malignant B cells in the bone marrow, lymph nodes, and peripheral blood [42]. CLL patients usually suffer from secondary immunodeficiency due to hypogammaglobulinemia and abnormal cellular immunity [43]. In CLL, circulating malignant B cells deleteriously affect the T cell anti-tumor immunity [44, 45]. CLL-associated mortalities are mainly due to disease progression, secondary solid malignancy, and/or infections [46] that are governed by the compromised immune system in predisposed patients [47, 48]. T cell impairment/exhaustion is one aspect of the compromised anti-tumor immunity in CLL patients. Unfortunately, current immunotherapies targeting PD-1 and CTLA-4 pathways have not been encouraging in CLL patients [49]. This might be related to the differential nature of exhausted T cells in hematological cancers versus solid tumors. For example, we have shown that CD160, not PD-1is the dominant co-inhibitory receptor associated with CD8^+^ T cell exhaustion in CLL patients [45]. These examples provide an urgent need for a better understanding of T cell repertoire in CLL patients. Although CAR T cell therapy has been associated with promising results in CLL patients [50], further T cell-related studies will assist us in identifying potential novel immunotherapies.

In this study, we investigated the frequency of CD26^+^CD8^+^ T cells in a cohort of CLL patients in comparison with age-sex-matched healthy controls (HCs). We further performed extensive immunophenotyping on CD26^neg^, CD26^low^, and CD26^high^ CD8^+^ T cell subsets in both cohorts. Moreover, we conducted comprehensive studies on the effector functions of different subpopulations of CD26^+^CD8^+^ T cells in CLL versus HCs. Notably, we investigated the mechanism underlying the depletion of CD26^+^ T cells in CLL patients. Therefore, our studies provide a novel insight into the role of CD26^+^CD8^+^ T cells in CLL patients and suggest that CD26^high^CD8^+^ T cells may have promising potential for adoptive T cell transfer and CAR T cell therapies.

## Material and methods

### Study population

We recruited 55 patients with confirmed CLL for this study (Additional file 2: Table S1), along with 44 age-and-sex-matched healthy controls for comparison. We collected peripheral blood specimens and bone-marrow aspirates in EDTA-containing tubes. The clinical data including IGHV mutation status, FISH analysis, clinical staging (Rai staging system) [51], and treatment state/course were collected for further analysis.

### Cell isolation and purification

The peripheral blood mononuclear cells (PBMCs) and the bone-marrow cells were isolated using Ficoll-Paque gradients (GE Healthcare). CD3^+^ T cells were enriched by a negative selection kit (EasySep isolation kit, Stem Cell Technologies) with a purity of > 97% (Additional file 1: Fig. S1a). For effector T cell (CD3^+^CCR7^−^) isolation, CD3^+^ T cells were stained with the PE-conjugated anti-CCR7 antibody followed by the anti-PE-conjugated microbeads (Miltenyl) with a purity of > 96% (Additional file 1: Fig. S1b). For B cell enrichment, B cells were stained with FITC-conjugated anti-CD19 antibody and then isolated by the anti-FITC microbeads (Miltenyl) with a purity of > 91% (Additional file 1: Fig. S1c).

### Flow cytometry

The fluorochrome-conjugated antibodies were purchased from BD Biosciences, Thermo Fisher Scientific or Biolegend including human anti-CD3 (SK7), anti-CD4 (RPA-T4), anti-CD8 (RPA-T8), anti-CD26 (M-A261), anti-CD161(HP-3G10), anti-TV $\alpha$ 7.2 (3C10), anti-IL-18R $\alpha$ (H44), anti-CD5 (UCHT2), anti-CD19 (HIB19), anti-CD160(BY55), anti-2B4(eBioDM244), anti-TIGIT(MBSA43), anti-PD1 (EH12.1), anti-TIM-3 (7D3), anti-CD39 (TU66), anti-CD73 (AD2), anti-CD95 (DX2), anti-CD127 (HIL-7R-M21), anti-ROR $\gamma \delta$(Q21-559), anti-CD45RA (HL100), anti-CCR7 (3D12), anti-CD27 (G3H69), anti-CD28 (CD28.2), anti-ICOS (C398.4A), anti-CD57 (NK-1), anti-CD16 (B73.1), anti-CD56 (B159), anti-KLRG1 (2F1/KLRG1), anti-CD69 (N50), anti-CD107a (H4A3), anti-IL-2 (MQ1-17H12), anti-TNF-$\alpha$(MAB11), anti-IFN-$\gamma$(45.B3), anti-Perforin (dG9), anti-Granzyme-B (GB11), anti-Granzyme-K (G3H69), anti-CLA (HECA452, Miltenyi), anti-CCR4 (1G1), anti-CCR5 (2D7/CCR5), anti-CCR6 (11A9), anti-Integrin-$\beta$ 7 (FIB504), anti-CXCR3 (1C6/CXCR3), anti-CXCR4 (12G5), anti-Galectin-9 (9M1-3), anti-Annexin-V (Annexin V), anti-TOX (TXRX10), anti-FOXP3 (150D/E4), and anti-T-bet (4B10). We also used mouse anti-CD26 (H194-112), anti-CD8 (53–6.7), and anti-CD3 (17A2) antibodies.

Surface staining was performed, as we have reported elsewhere [52, 53]. Data were acquired on an LSR Fortessa-SORP (BD Bioscience) and subsequently analyzed using Flow Jo software (V.10.8.1). Cell viability was analyzed using the LIVE/DEAD kit (Life technologies).

### Cell culture and ex vivo cytokine measurement

Isolated PBMCs were cultured and stimulated with soluble Purified NA/LE anti-human CD3 (UCHT1, 3 μg/ml)/CD28 (CD28.2, 1 μg/ml) or PMA (20 ng/ml)/Ionomycin (1 g$\mu$/ml) (Cell stimulation cocktail, Biolegend) in the presence of the protein transport inhibitor Brefeldin A (BD Biosciences, 1/1000) for 5 h. Intracellular cytokine staining was performed according to our protocols [54]. For cytokine-dependent cultures, PBMCs were treated with a cocktail of cytokines including recombinant human IL-12 (Cedarlane,100 ng/ml), IL-18 (Biolegend,100 ng/ml), and IL-15 (Biolegend, 100 ng/ml) for 18 h. Brefeldin A (1/1000) was added 5 h before the intracellular staining. In other experiments, the effects of different cytokines including TNF-α (50 ng/ml), IFN-γ (100 ng/ml), IL-10 (100 ng/ml), IL-16 (500 ng/ml), IFN-α (100 ng/ml), IL-2 (20 ng/ml), IL-6 (100 ng/ml), and TGF-β (20 ng/ml) on CD26 expression was analyzed.

### qPCR analysis

The RNA was isolated from CD8^+^ T cells from HCs and CLL patients using the Direct-zol RNA MicroPrep kit (Zymo Research). cDNA was synthesis using the Quantitect Reverse Transcription Kit (Qiagen). RT-PCR was carried out using the Quantitect primer Kit (Qiagen) to measure the expression of CD26 mRNA. Each sample was run in duplicate, using the CFX96 Touch Real-Time PCR Detection System (BioRad). Beta-2-microglobulin was used as a reference gene and the relative fold change of the targeted genes was calculated by the ΔΔ CT method.

### Proliferation assay

Isolated effector T cells (CD3^+^ CCR7^−^) were labeled with the CFSE dye (Life Technologies) before stimulation using the Dyna beads Human T-activator CD3/CD28 (Thermo Fisher Scientific) according to the manufacturer’s instruction and our protocols [53, 54]. After 72 h cells were stained and analyzed.

### Migration assay

The migration assay was performed using the CytoSelect migration assay kit (Cell Biolabs), as we have reported elsewhere [55, 56]. PBMCs were starved overnight in FBS-free culture media. The next day, FBS (10%), recombinant human RANTES (CCL5) (R and D, 10 nM), and recombinant human IL-18 (Biolegend, 100 ng/ml) were used as chemoattractants. Cell suspension of starved cells (0.5 × 10^6^ cells/well) was added to the upper chamber and incubated in the incubator (37 °C, 5% CO25) for 24 h. Migrated cells in the lower chamber were harvested and quantified by flow cytometry according to the manufacturer’s instructions. The migration ratio was calculated compared to the wells lacking the chemoattractant.

### Multiple and ELISA assay

The plasma concentration of cytokines/chemokines was measured using the Meso Scale Discovery (MSD) multiplex kit, as we have reported elsewhere [45]. Data were acquired on the V-plex^®^ Sector Imager 2400 plate reader and analyzed using the MSD Workbench 3.0 software. In addition, soluble CD26, IL-18, TGF-β, and Galectin-9 (Gal-9) were detected using the DuoSet ELISA kit (R&D) according to the manufacturer’s protocol. The microplate reader (Synergy H1 Biotek) was used for acquiring ELISA data and analyzed by Gen5 V.2.07 software.

### Statistical analysis

GraphPad Prism software (version 9.3.1) was used for statistical analysis. Mann–Whitney U test or Wilcoxon signed rank test was used for non-paired or paired comparisons, respectively. For multiple comparisons, the Kruskal–Wallis one-way analysis of variance test was used. Data were presented as median with an interquartile range. *P-*values less than 0.05 was considered statistically significant. The visual summary was prepared using the BioRender software.

## Results

### A significant reduction in CD26^+^CD8^+^ T cells in CLL patients

**Fig. 1:**
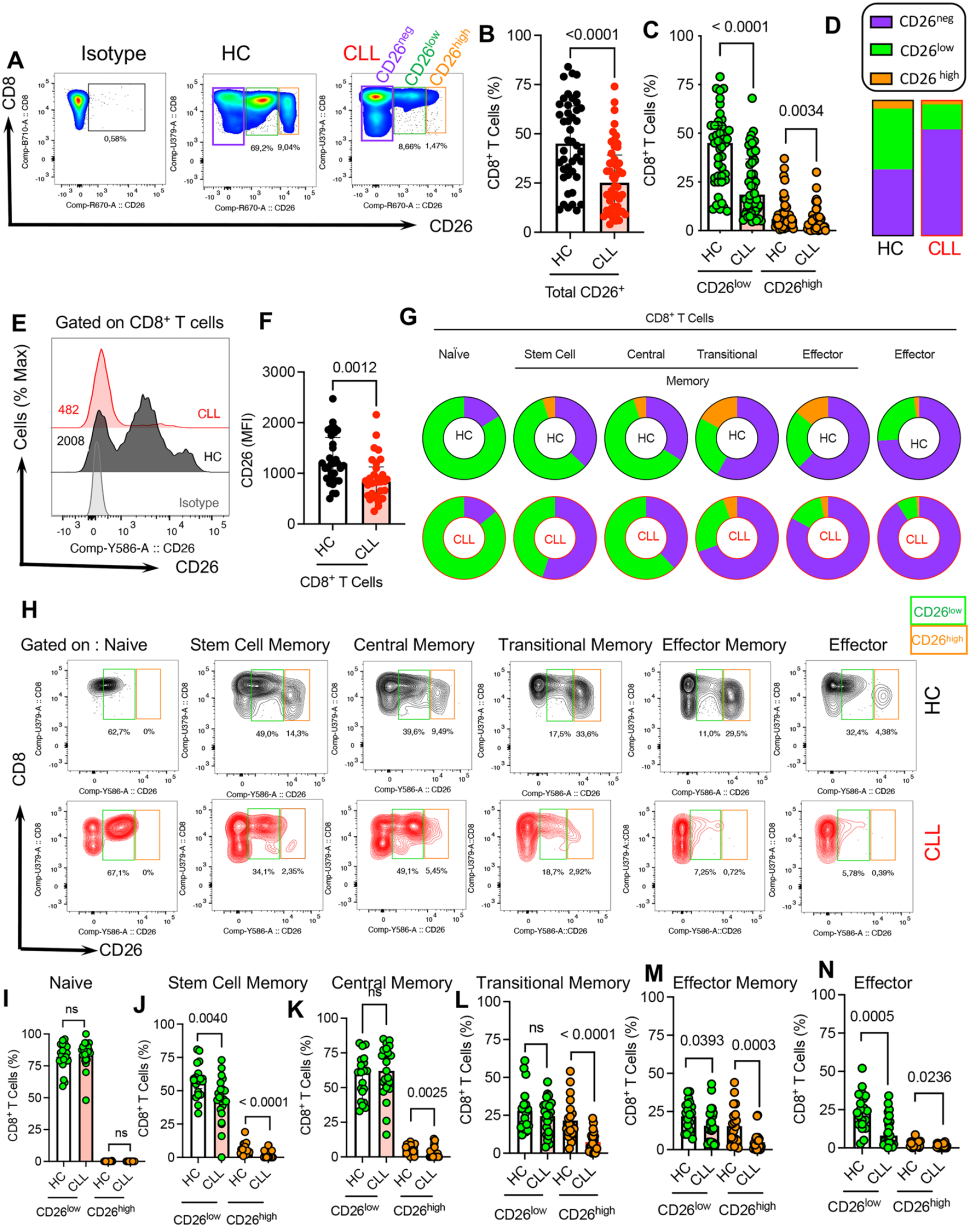
CD26^low^ and CD26^high^ CD8^+^ T cells are reduced in CLL. **A** Representative flow cytometry plots, and (**B**) Cumulative data comparing the frequency of CD26^+^CD8^+^ T cells in PBMCs from HCs (n = 44) and CLL (n = 55) patients. **C** Cumulative data comparing the frequency of CD26^low^ and CD26^high^ CD8^+^ T cells in HC and CLL. **D** Bar plots illustrating the proportion of CD26^neg^, CD26^low^ and CD26^high^CD8^+^ T cells in HC and CLL (**E**) Representative histogram plots, and (**F**) Cumulative data comparing the Mean Fluorescence Intensity (MFI) of CD26 in CD8^+^ T cells in HCs and CLLs. **G** The pie charts represent the median frequency of CD26^neg/low/high^ in different subsets of CD8^+^ T cells (e.g. Naïve, Stem cell memory, Central memory, Transitional memory, Effector memory, and Effector) in HCs versus CLLs. **H** Representative flow cytometry plots of CD26^low/high^ in different CD8^+^ T cell subsets of HCs (black) and CLLs (red). **I** Cumulative data of CD26^low/high^ in naïve, (**J**) stem cell memory, (**K**) central memory, (**L**) transitional memory, (**M**) effector memory, and (**N**) effector CD8^+^ T cells in HC and CLL. Statistics are assessed by Mann–Whitney or the Kruskal–Wallis multiple comparison tests. P-value < 0.05 was considered as significant. Error bars represent the median with an interquartile range. Each dot represents an individual human sample

To determine the frequency of CD26^+^CD8^+^ T cells, PBMCs from CLL patients (n = 55) and healthy controls (HCs) (n = 44) were subjected to CD26 expression analysis. (Additional file 1: Fig. S1d, the gating strategy). These studies revealed that the frequency of CD26^+^CD8^+^ T cells was significantly declined in CLL patients compared to HCs (Fig. 1A, B). While on average half of CD8^+^ T cells in PBMCs of HCs expressed CD26 (Mean $\pm$ SD: 45.88 $\pm$ 21.36) this was substantially lower (Mean $\pm$ SD: 26.84 $\pm$ 16.64) in CLL (Fig. 1A, B). Although the majority of CD26 expressing CD8^+^ T cells were CD26^low^, the proportions of both CD26^high^ and CD26^low^ were significantly reduced in CLL patients compared to HCs (Fig. 1A, C, D). We also measured the cell number in both groups, which confirmed a significant reduction in the number of CD26^low^ and CD26^high^ T cells in CLL patients (Additional file 1: Fig. S1e). Moreover, the intensity of CD26 expression was significantly decreased in CD8^+^ T cells of CLL patients compared to HCs (Fig. 1E, F). As expected, the CD26^low^ subpopulation had a significantly lower intensity of CD26 expression than their CD26^high^ counterparts in CLL patients (Additional file 1: Fig. 1f).

Given the reported impact of age and sex on T cell repertoire [57], we found these variables did not influence the frequency of CD26^+^CD8^+^ T cells (Additional file 1: Fig. S1g, h). Also, we did not observe any difference in the frequency of CD26^+^CD8^+^ T cells among treated (n = 18) versus untreated (n = 37) CLL patients (Additional file 1: Fig. S1i, j). To determine the potential correlation between the clinical staging of CLL patients with the frequency of CD26^+^CD8^+^ T cells, we stratified our patients according to the Rai staging system for CLL into three groups [51]; however, we did not observe any significant difference between them (Additional file 1: Fig. S1k). Moreover, our analysis revealed that the lymphocyte count in the whole blood did not correlate with the frequency of CD26^+^CD8^+^ T cells (Additional file l Fig. S1l). To determine whether CD26 downregulation was CD8^+^ T cells specific or a general phenomenon of CLL, we measured the expression of CD26 on other blood mononuclear cells. Although CD4^+^ T cells were the most dominant CD26 expressing cells, their frequency in CLL patients was also significantly reduced compared to HCs (Additional file 1: Fig. S1m–o). Of note, NK cells exhibited a very small proportion of CD26-expressing cells without any difference between HCs and CLL patients (Additional file 1: Fig. S1p). Also, we compared the mean fluorescence intensity (MFI) of CD26 in malignant B cells in CLL with non-malignant B cells. These studies revealed a significant increase in the intensity of CD26 in malignant B cells, which is consistent with previous reports [58, 59] (Additional file 1: Fig. S1q). These observations suggested that CD26 may get shed from the cell surface resulting in the elevation of soluble CD26 in the plasma. However, the plasma levels of CD26 in CLL patients did not support this hypothesis (Additional file 1: Fig. S1r). Moreover, we compared CD26^+^CD8^+^ frequencies in the bone marrow and blood of CLL patients. Despite a trend towards lower CD26^+^CD8^+^ T cells in the bone marrow, it was not significant (Additional file 1: Fig. S1s, t). Finally, to understand the stage of CD26 reduction, we quantified CD26 mRNA levels in CD8^+^ T cells from CLL and HCs. However, we did not find any significant difference between the groups at the gene level (Additional file 1: Fig. S1u). Overall, these observations support the notion that CLL is associated with a substantial decline in the frequency of CD26^+^ T cells, particularly CD26^low^CD8^+^ T cells, without any changes in the plasma levels of soluble CD26.

### The differential expression pattern of CD26 in CD8^+^ T cell subsets in CLL

To better phenotype CD26^+^CD8^+^ T cells in CLL, we conducted a detailed ex vivo analysis of these cells. Based on CD45RA, CCR7, CD95, and CD27 markers, we characterized T cell subsets such as naïve (CD45RA^+^CCR7^+^CD95^−^), stem cell memory (CD45RA^+^CCR7^+^CD95^+^), central memory (CD45RA^−^CCR7^+^), transitional memory (CD45RA^−^CCR7^−^ CD27^+^), effector memory (CD45RA^−^CCR7^−^ CD27^−^), and effectors (CD45RA^+^CCR7^−^) [60–62]. We found that CD8^+^CD26^low^ T cells were mainly naïve, stem cell memory, and central memory (Fig. 1G). As illustrated in this figure, the frequency of CD8^+^CD26^low^ expressing T cells declines as T cells differentiate to other subsets (e.g. transitional memory, effector memory, and effectors) in both HCs and CLL patients. In contrast, CD8^+^CD26^high^ T cells were uniquely populated in transitional and effector memory subsets with very low frequency in other subsets and absent in the naïve population (Fig. 1G). In particular, we observed that the frequency of CD26^low^ was significantly lower in stem cell memory, effector memory, and effectors but unchanged in other T cell subsets in CLL patients compared to HCs (Fig. 1H–N). However, the frequency of CD26^high^CD8^+^ T cells was significantly lower in all T cell subsets except the naïve subset in CLL patients compared to HCs (Fig. 1H–N). Despite the expansion of total effector memory and effector CD8^+^ T cell subsets in CLL patients (Additional file 1: Fig. S2a, b), the frequency of those expressing CD26^high^ was significantly lower in CLL patients (Fig. 1M, N).

Moreover, to better characterize CD26^+^CD8^+^ T cells, we subjected them to CD27 expression analysis. CD27 is involved in CD8^+^ T cell activation and memory formation that augments anti-tumor activity [63, 64]. Our further studies confirmed the abundance of transitional and effector memory subsets in CD26^low^ and CD26^high^CD8^+^ T cells (Additional file 1: Fig. S2c, d).

Overall, our results indicate that CD26^+^CD8^+^ T cells are in distinct stages of differentiation. Considering the substantial co-expression of CD27 and CD26, a considerable decline in the proportion of CD26^+^CD8^+^ T cells may deprive CLL patients of the potent anti-tumor activity of this T cell subset [64].

### CD26^high^CD8^+^ T cells are enriched with MAIT cells in CLL

**Fig. 2:**
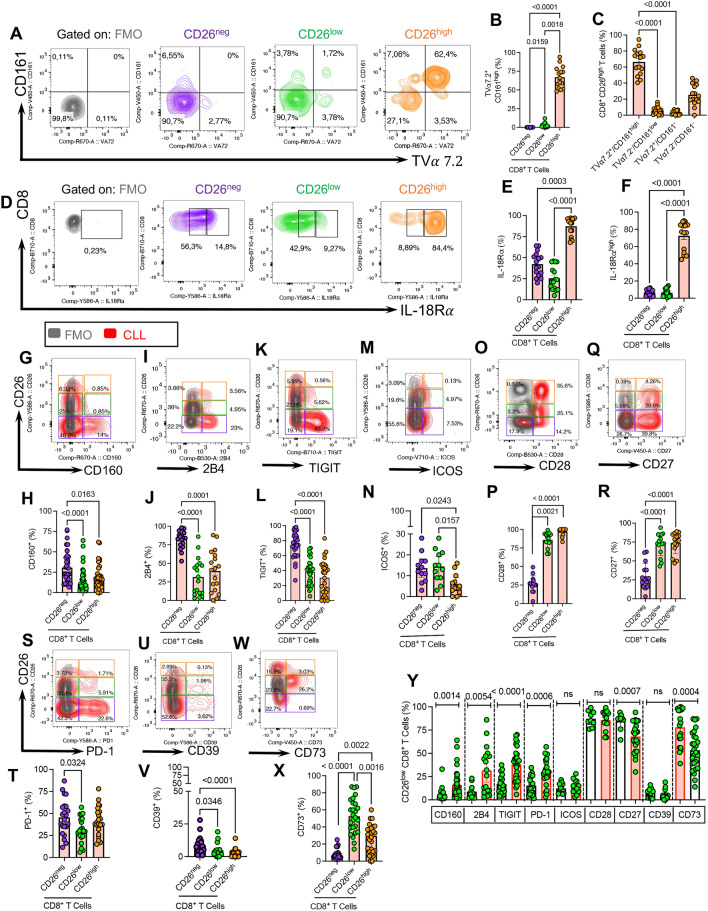
Phenotypic profile of CD26^neg/low/high^ CD8^+^ T cells in CLL. **A** Representative flow plots, and (**B**) cumulative data showing the frequency of CD161^+^ TV $\alpha$ 7.2^+^ in CD26^neg/low/high^ CD8^+^ T cell subsets in CLL. **C** Cumulative data of the frequency CD161/ TV $\alpha$ 7.2 co-expressing cells in CD26^high^CD8^+^ T cells in CLL. **D** Representative flow plots, and (**E**) cumulative data of the frequency of IL-18R $\alpha$ expressing cells among CD26^neg/low/high^ CD8^+^ T cells in CLL. **F** Cumulative data of the frequency of IL-18R $\alpha$ ^high^ expressing cells among CD26^neg/low/high^ CD8^+^ T cells in CLL. **G** Representative plots, and (**H**) the frequency of CD160^+^ cells among CD26^neg/low/high^ CD8^+^ T cells in CLL. **I** Representative plots, and (**J**) cumulative data of the frequency of 2B4^+^ cells among CD26^neg/low/high^ CD8^+^ T cells in CLL. **K** Representative plots, and (**L**) cumulative data of the frequency of TIGIT^+^ cells among CD26^neg/low/high^ CD8^+^ T cells in CLL. **M** Representative plots, and (**N**) cumulative data of the frequency of ICOS^+^ cells among CD26^neg/low/high^ CD8^+^ T cells in CLL. **O** Representative plots, and (**P**) cumulative data of the frequency of CD28^+^ among CD26^neg/low/high^ CD8^+^ T cells in CLL. **Q** Representative plots, and (**R**) cumulative data of the frequency of CD27^+^ cells among CD26^neg/low/high^ among CD8^+^ T cells in CLL. **S** Representative plots, and (**T**) cumulative data of the frequency of PD-1^+^ cells among CD26^neg/low/high^ CD8^+^ T cells in CLL. **U** Representative plots, and (**V**) cumulative data of the frequency of CD39^+^ cells among CD26^neg/low/high^ among CD8^+^ T cells in CLL. **W** Representative plots, and **X** cumulative data of the frequency of CD73^+^ cells among CD26^neg/low/high^ CD8^+^ T cells in CLL. **Y** Cumulative data showing the frequency of co-inhibitory/co-stimulatory expressing cells in CD26^low^CD8^+^ T cells in HC and CLL. Error bars represent the median with an interquartile range. Each dot represents an individual human sample. Florescence minus one (FMO)

MAIT cells express high levels of CD161 [40], IL-18R $\alpha$, and CD26 [36, 41]. In particular, the most specific surrogate marker for MAIT cells is the co-expression of CD161^high^ and TV $\alpha$ 7.2 [65]. As such, we decided to determine whether CD26^+^CD8^+^ T cells were MAIT cells. We observed that the majority (Mean $\pm$ SD: 67 $\pm 14.48$) of CD26^high^CD8^+^ T cells co-expressed TV $\alpha$ 7.2^+^ & CD161^high^ in CLL patients (Fig. 2A–C). However, a portion of CD26^high^ did not express TV $\alpha$ 7.2 and CD161^high^ (Fig. 2A, C). Of note, the frequency of MAIT-like cells expressing TV $\alpha$ 7.2 and CD161^high^ was significantly lower among CD8^+^CD26^+^ T cells in CLL patients (Additional file 1: Fig. S2e–g). Moreover, we investigated the expression levels of IL-18R $\alpha$ in three subsets of CD26^neg^, CD26^low^, and CD26^high^CD8^+^ T cells, which elucidated that CD26^high^ cells were the dominant cells expressing IL-18R $\alpha$ in both HCs and CLL patients. However, a portion of CD26^high^ CD8^+^ T cells lacked the expression of this cytokine receptor (Fig. 2D–F). These observations suggest that CD26^high^CD8^+^ T cells are enriched with MAIT-like cells but they are a heterogeneous T cell subset. It is worth mentioning that we found a considerable frequency of MAIT-like cells that did not express CD26^high^. As such, we speculate that CLL may impact the expression of MAIT surrogate markers. Therefore, CD26^high^ might not be a definite marker for MAIT cell identification in CLLs. However, the majority of CD26^high^CD8^+^ T cells displayed the MAIT-like phenotype (CD161^high^ TV $\alpha$ 7.2^+^) and significantly declined in CLL patients compared to HCs.

### The heterogeneous expression of co-inhibitory and co-stimulatory receptors in CD26^+^CD8^+^ T cells

To better characterize CD26^+^ versus their negative counterparts, we subjected them to further analysis for the expression of co-stimulatory/inhibitory molecules. Previously, we have reported that the co-inhibitory receptor, CD160, was selectively overexpressed on CD8^+^ T cells in CLL patients [45]. In agreement, we found that CD26^neg^CD8^+^ T cells were significantly enriched with CD160 (Fig. 2G, H), 2B4 (Fig. 2I, J), TIGIT (Fig. 2K, L), and ICOS (Fig. 2M, N) expressing T cells than their CD26^low^ and CD26^high^ siblings. On the contrary, CD26^low^ and CD26^high^ T cells were significantly populated with CD28 and CD27 expressing CD8^+^ T cells (Fig. 2O–R). Interestingly, we did not observe any difference in the proportion of PD-1 expressing CD8^+^ T cells between CD26^neg^, CD26low, and CD26^high^ subsets ((Fig. 2S, T). Although HCs, in general, have a lower frequency of T cells expressing co-inhibitory/stimulatory receptors, we observed a significant reduction in the proportion of CD160, 2B4, and PD-1 expressing cells in CD26^low^CD8^+^ T cell subset compared to their CD26^neg^ or CD26^high^ siblings (Additional file 1: Fig. S2h–j). The frequency of TIGIT-expressing T cells was significantly lower in CD26^low^/CD26^high^ subsets in HCs (Additional file 1: Fig. S2k). Although ICOS and CD28 had similar expression patterns in CD26^−/+^ subsets, CD27^+^CD8^+^ T cells were significantly abundant in the CD26^low^ subset in HCs (Additional file 1: Fig. S2l–n). Owing to the tandem contribution of ectonucleotidases CD39, CD73, and CD26 in the adenosine pathway [66], we measured the expression of these two ectoenzymes in different subsets of CD26-expressing CD8^+^ T cells. We found that CD39 was highly expressed in CD26^neg^CD8^+^ T cells (Fig. 2U, V), whereas CD73 was prominently expressed in CD26^low^ followed by CD26^high^ and CD26^neg^ CD8^+^ T cells (Fig. 2W, X). Similarly, we analyzed the expression of CD39 and CD73 in CD26^+^CD8^+^T cells of HCs, which showed no differential expression pattern for CD39 but we found a higher abundance of CD73^+^ T cells among CD26^low^ compared to their CD26^neg^/CD26^high^ counterparts (Additional file 1: Fig. S2o, p). Our further analysis revealed that CD26^low^CD73^+^ T cells predominantly displayed naïve T cell phenotype (Additional file 1: Fig. S2q, r). However, the subpopulation of CD26^+^CD73^+^ with effector and effector memory phenotype (CD8^+^CCR7^−^ T cells) was significantly reduced in CLL patients versus HCs (Additional file 1: Fig. S2s). Overall, we found that CD160, 2B4, TIGIT, and PD-1 expressing CD26^low^CD8^+^ T cells were significantly enriched in CLL versus HCs (Fig. 2Y). In contrast, the proportion of CD27^+^CD73^+^ expressing cells was significantly reduced in the CD26^low^CD8^+^ T cell subset without any changes in the frequency of ICOS, CD28, CD39 expressing CD8^+^ T cells in CLL versus HCs (Fig. 2Y). However, this pattern was different for CD26^high^ T cells and they showed higher expression of TIGIT^+^ and CD27^+^ cells in CLL patients compared to HCs (Additional file 1: l Fig. 2t). Interestingly, we found that the proportion of 2B4^+^ T cells was significantly increased but CD28, CD27 and CD73-expressing cells were decreased among CD26^neg^CD8^+^ T cells in CLL patients (Additional file 1: Fig. 3a).

### CD26^neg^CD8^+^ T cells exhibit higher cytotoxic properties

**Fig. 3:**
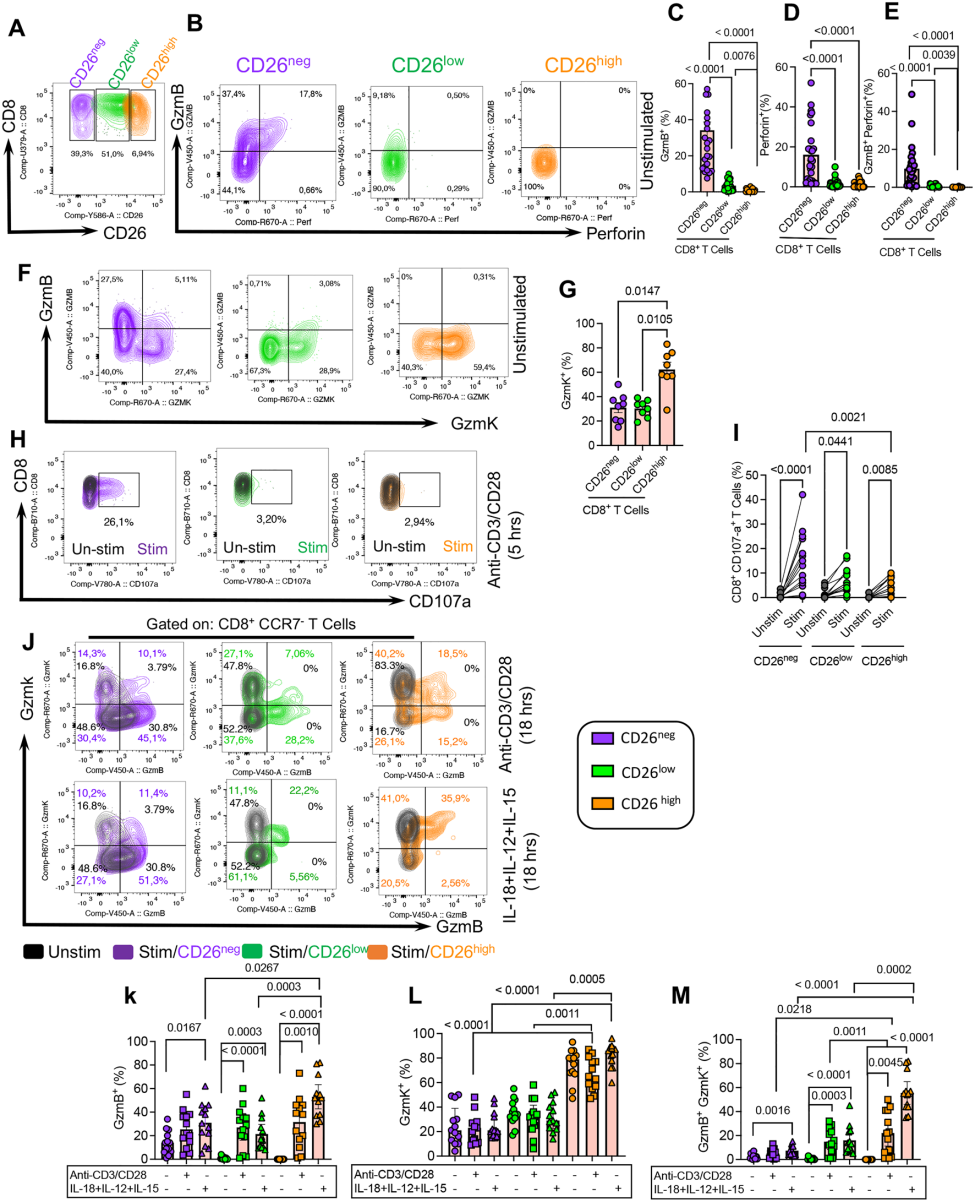
Cytotoxic properties of CD26^neg/low/high^ CD8^+^ T cells in CLL. **A** Representative flow plot of the gating strategy for CD26^neg^, CD26^low^, and CD26^high^ CD8^+^ T cells. **B** Representative flow plots of the co-expression of Granzyme-B (GzmB)/Perforin in CD26^neg/low/high^ CD8^+^ T cells in CLL. **C** Cumulative data showing the frequency of GzmB^+^, (**D**) Perforin^+^, and (**E**) GzmB^+^Perforin^+^ cells among CD26^neg/low/high^ CD8^+^ T cells in CLL. **F** Representative flow plots, and (**G**) cumulative data of the frequency of GzmB^+^GzmK^+^ cells among CD26^neg/low/high^ CD8^+^ T cells in CLL. **H** Representative flow plots, and (**I**) cumulative data of the frequency of CD107a^+^ cells among CD26^neg/low/high^ CD8^+^ T cells in CLL either unstimulated (unstim) or stimulated (stim) with anti-CD3/CD28 (3 g$\upmu$/ml, 1 g$\upmu$/ml) in the presence of protein transport inhibitor (1/1000). **J** Representative flow plots, and cumulative data of the frequency of (**K**) GzmB^+^ (**L**) GzmK^+^, and (**M**) GzmB^+^GzmK^+^ cells among CD26^neg/low/high^ CCR7^−^CD8^+^ T cells in CLL either unstimulated or stimulated with anti-CD3/CD28(3 g$\upmu$/ml, 1 g$\upmu$/ml), and a cocktail of IL-18 + IL-12 + IL-15 (100 ng/ml of each). Error bars represent median with interquartile range. Each dot represents an individual human sample

CD8^+^ T cells as cytotoxic T lymphocytes play an essential role against virally infected and tumor cells [67, 68] via granule-mediated cytotoxicity, FAS-FASL interaction, and the release of cytokines (e.g. TNF-α and IFN-γ) [69]. Granzymes as cytolytic molecules and their harmonized action with perforin (pore forming protein) are required for effective granule-mediated cytotoxicity [70]. To determine the granule-mediated cytotoxic ability of CD26^±^CD8^+^ T cells, we measured the intracytoplasmic expression of perforin and granzyme-B (GzmB) in CD26^neg^, CD26^low^, and CD26^high^ CD8^+^ T cells in CLL patients ex vivo. We found that in contrast to the CD26^neg^ subset, CD26^low^ and CD26^high^ cells were devoid of perforin and expressed very low levels of GzmB (Fig. 3A–E). Considering the heterogeneous nature of CD8^+^ T cells, we observed that even CD26^low/high^CD8^+^ T cells with effector and effector memory phenotype showed substantial downregulation of perforin/GzmB compared to their CD26^neg^ counterparts in CLL patients (Additional file 1: l Fig. S3b–d). We found the same phenotype in terms of GzmB and perforin expression in CD26^low/high^CD8^+^ T cells in HCs (Additional file 1: Fig. S3e–g). To better characterize the potential cytolytic role of CD26^+^CD8^+^T cells, we subjected them to GzmK expression analysis. Interestingly, we observed that CD26^high^CD8^+^ T cells expressed substantial levels of intracytoplasmic GzmK compared to CD26^low^ and CD26^neg^ T cells (Fig. 3F, G). Moreover, we assessed the degranulation capacity of CD8^+^ T cells by measuring CD107a expression (Lysosomal-associated membrane protein I (LAMP-I)) [71] in response to the global stimulation with anti-CD3/CD28 antibodies. These studies revealed that CD26^neg^CD8^+^ T cells had higher degranulation capacity following in vitro stimulation than CD26^low/high^ CD8^+^ T cells (Fig. 3H, I). To characterize the functional properties of CD26^+^CD8^+^ T cells in response to stimulation, we stimulated them either via T Cell receptor (TCR) (anti-CD3/CD28) or a cytokine cocktail (IL-18 + IL-12 + IL-15) for 18 h. We found that TCR-mediated stimulation significantly increased GzmB expression in CD26^low^ and CD26^high^ CD8^+^ T cell populations, while GzmK expression levels remained unchanged (Fig. 3J–M). Of note, TCR stimulation did not change the expression levels of GzmB and GzmK in CD26^neg^ CD8^+^ T cells (Fig. 3J–M). However, cytokine-mediated stimulation significantly enhanced GzmB/GzmK co-expression in all T cell subsets, but was more pronounced in the CD26^high^CD8^+^ T cell subset (Fig. 3J–M). The same expression pattern was observed for the upregulation of perforin in different T cell subsets after stimulation (Additional file 1: Fig. S3h, i). Overall, these observations revealed differential expression of GzmB and GzmK in CD26^±^CD8^+^ T cell subsets in CLL patients. Collectively, while CD26^neg^CD8^+^ T cells exhibited higher GzmB and perforin expression at the baseline, CD26^high^ and CD26^low^ CD8^+^ T cells acquired a greater cytolytic molecules expression upon stimulation.

### CD26^high^CD8^+^ T cells display higher cytokine-induced responsiveness

**Fig. 4:**
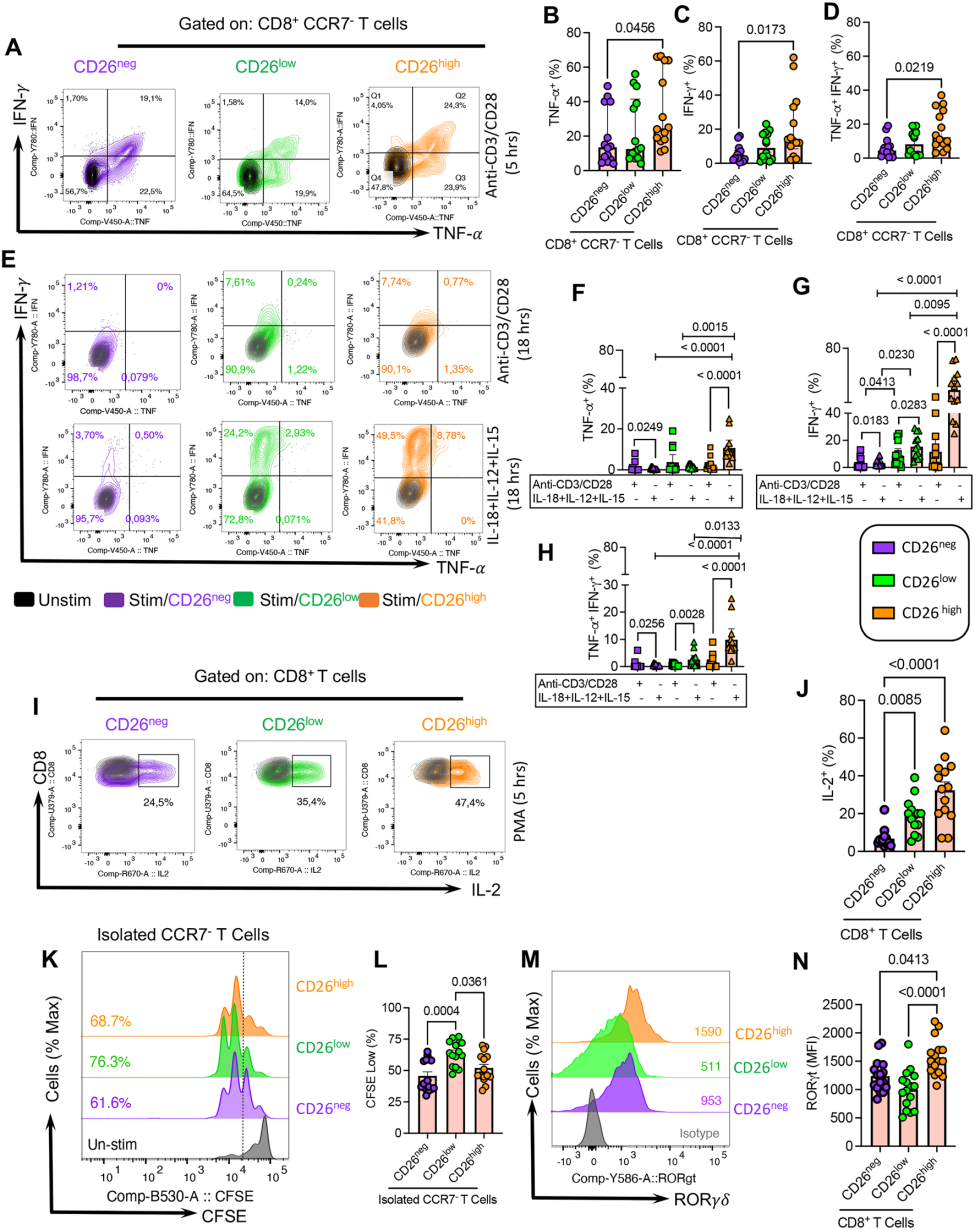
Cytokine production and proliferation ability of CD26^neg/low/high^ CD8^+^ T cells in CLL. **A** Representative flow plots, (**B**) the frequency of TNF-$\alpha$, (**C**) IFN-$\gamma ,$ and (**D**) TNF-$\alpha$^+^IFN-$\gamma$^+^ cells among CD26^neg/low/high^ CCR7^−^CD8^+^ T cells. **E** Representative plots, (**F**) cumulative data showing the frequency of TNF-$\alpha$^+^, (**G**) IFN-$\gamma$^+^, and (**H**) TNF-$\alpha$^+^IFN-$\gamma$^+^ among CD26^neg/low/high^ CCR7^−^CD8^+^ T cells in unstimulated or stimulated with anti-CD3/CD28 (3 g$\upmu$/ml, 1 g$\upmu$/ml) and a cocktail of IL-18 + IL-12 + IL-15 (100 ng/ml of each). (**I**) Representative flow plots and, (**J**) cumulative data of the frequency of IL-2 expressing cells among CD26^neg/low/high^ CD8^+^ T cells in unstimulated (black color) versus 5 h after *in-vitro* stimulation with PMA/ionomycin cocktail (Biolegend, 2 ng/ml) in the presence of Brefeldin A (1 g$\mu$/ml). (**K**) Representative flow plots and, (**L**) cumulative data of the frequency of CFSE^low^ (proliferated) cells among CD26^neg/low/high^ CCR7^−^CD8^+^ T cells, unstimulated (black color) versus 72 h stimulation. (**M**) Representative flow plots, and (**N**) cumulative data showing the MFI for ROR $\gamma \delta$ in CD26^neg^, CD26^low^, and CD26^high^ CD8^+^ T cells in CLL. Error bars represent the median with an interquartile range. Each dot represents an individual human sample

To better delineate the effector functions of CD26^+^CD8^+^ T cells, we analyzed their cytokine production capacity (e.g., IFN-$\gamma$ and TNF-$\alpha$). We found that CD26^high^ exhibited significantly higher IFN-$\gamma$, TNF-$\alpha$, and IFN-γ/TNF-α expression than their CD26^neg^ counterparts following 5 h stimulation with anti-CD3/CD28 antibodies in vitro (Fig. 4A–D). However, stimulation of PBMCs with PMA for the same period induced higher IFN-$\gamma$, TNF-$\alpha$, and IFN-γ/TNF-α in CD26^neg^ and CD26^high^ compared to CD26^low^CD8^+^ T cells, while the magnitude of cytokine response was more pronounced in CD26^high^CD8^+^ T cells (Additional file 1: Fig. S3j–l). Next, we stimulated PBMCs via TCR-dependent (anti-CD3/CD28) or cytokine-dependent (IL-18 + IL12 + IL-15) manners for 18 h. Interestingly, we found that CD26^high^CD8^+^ T cells exhibited a greater cytokine production capacity than their other counterparts (e.g., CD26^neg^ and CD26^low^) within the same CLL patients (Fig. 4E–H).

We also measured IL-2 expression following 5 h PMA stimulation and found that CD26^high^ followed by CD26^low^ T cells had a superior capacity for IL-2 production compared to CD26^neg^ CD8^+^ T cells in CLL patients (Fig. 4I, J). However, we found that the proliferative capability of different CD8^+^ T cell subsets did not correspond with their cytokine production capacity. As such, CD26^low^CD8^+^ effector T cells displayed a significantly greater proliferation compared to their CD26^neg/high^ siblings following 72 h TCR stimulation in vitro (Fig. 4K, L). Moreover, we assessed the expression of cytokines in different CD8^+^ T cell subsets in HCs and noted that CD26^neg^ and CD26^high^ cells had higher levels of IFN-$\gamma$ and TNF-$\alpha$ compared to CD26^low^CD8^+^ T cells (Additional file 1: Fig. S3m–o).

Although CTLs are the best-characterized subpopulation of CD8^+^ T cells to kill infected cells or tumor cells, CD8^+^ T cells are highly heterogeneous. For example, it has been reported that environmental cues induce transcriptional factors to differentiate CD8^+^ T cells into Tc1, Tc2, Tc17, and Tc1/Tc17 cells that can be stratified based on the surface expression of CXCR3, CCR6, and CCR [72]. Therefore, we further characterized CD26^+^ and their CD26^neg^ counterparts in CLL patients, which showed CD26^high^ cells were enriched with Tc17 (CCR4^+^CCR6^+^) and Tc1/Tc17 (CCR6^+^CXCR3^+^) CD8^+^ T cell phenotype but Tc2 (CCR4^+^CCR6^−^) CD8^+^ T cells were more abundant in CD26^neg^ and CD26^low^ T cell subsets (Additional file 1: Fig. S3p–u). In addition, we evaluated the expression of different transcriptional factors in CD26-expressing subsets. We found a higher ROR $\gamma \delta$ expression [72] in CD26^high^ CD8^+^ T cells, which supports the Tc17 skewed phenotype of this T cell subset in CLL patients (Fig. 4m, n). However, we did not find any significant difference in T-bet and FOXP3 expression among CD26 subsets in CLL patients (Additional file 1: Fig. S4a, b). Interestingly, we noted a higher expression of TOX transcriptional factor in CD26^high^ than CD26^neg^ T cells in CLL patients (Additional file 1: Fig. S4c, d). Although TOX may be involved in T cell exhaustion, recent studies suggested that it is expressed by most effector memory/polyfunctional CD8^+^ T cells and not exclusively exhausted T cells in humans [73]. Therefore, our observations support this notion that TOX is not linked to exhaustion but polyfunctionality.

Overall, our observations support the heterogenous nature of CD26^high^CD8^+^ T cells with a greater cytokine production capacity compared to their CD26^neg/low^ counterparts.

### CD26^high^CD8^+^ T cells possess a greater migratory capacity

**Fig. 5:**
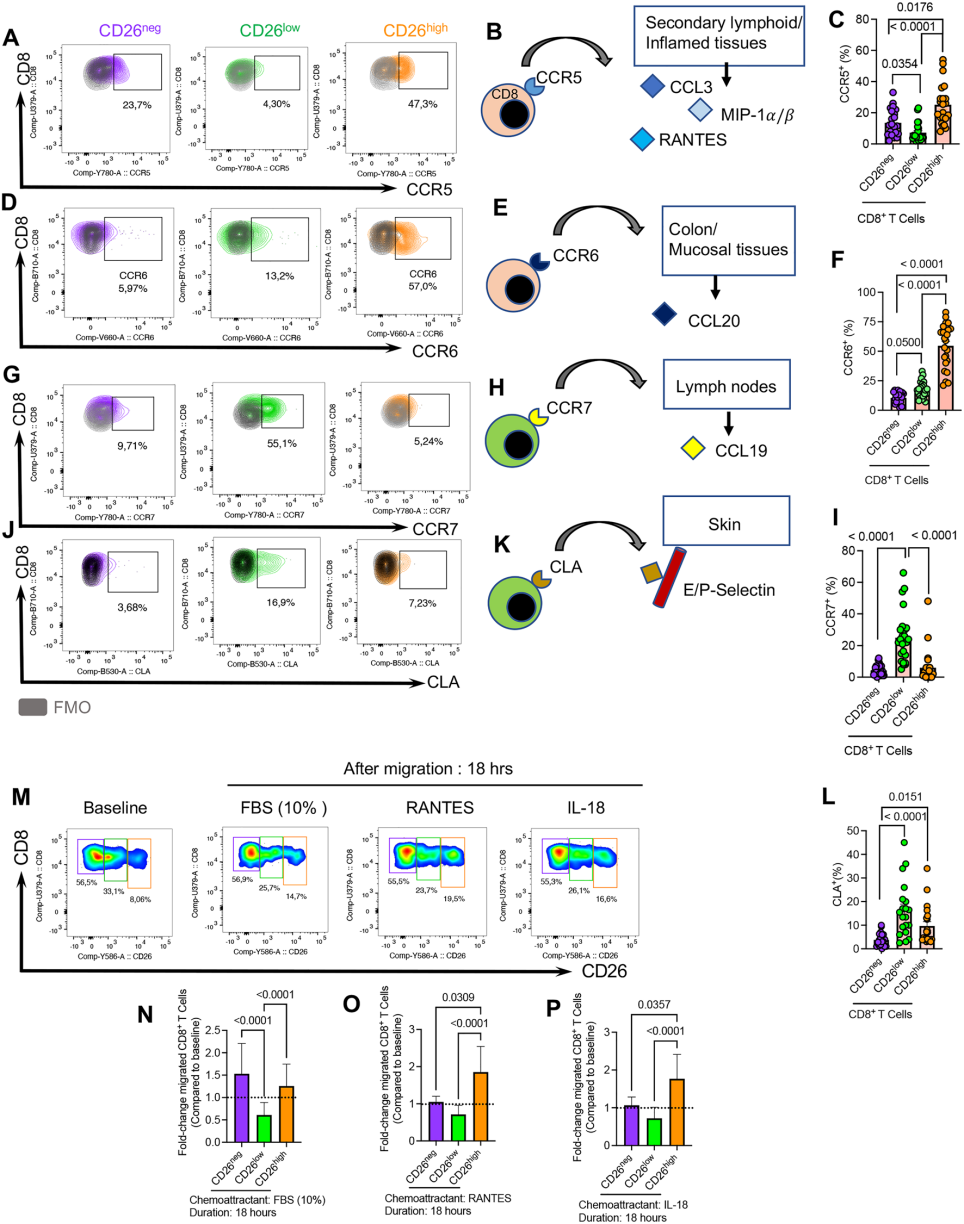
CD26^+^CD8^+^ T cells exhibit a greater migratory capacity in CLL. **A** Representative plots of the frequency of CCR5^+^CD8^+^ T cells among CD26^neg/low/high^ subsets. **B** The graphical illustrates the tendency of CCR5^+^CD8^+^ T cells homing to secondary lymphoid organs or inflamed tissues in response to CCL3, MIP-1 $\alpha$/$\beta$, and RANTES. **C** Cumulative data of the frequency of CCR5^+^CD8^+^ T cells among CD26^neg/low/high^ CD8 + T cells in CLL. **D** Representative flow plots of the frequency CCR6^+^CD8^+^ T cells among CD26^neg/low/high^ subsets. **E** The graphical illustrates the tendency of CCR6^+^CD8^+^ T cells homing to colon, and mucosal tissues in response to CCL20. **F** Cumulative data of the frequency of CCR6^+^CD8^+^ T cells among CD26^neg/low/high^. **G** Representative plots of the frequency of CCR7^+^CD8^+^ T cells among CD26^neg/low/high^ subsets of CD8^+^ T cells. **H** The graphical illustrates the tendency of CCR7^+^CD8^+^ T cells homing to lymph nodes in response to CCL19. **I** Cumulative data of the frequency of CCR7^+^CD8^+^ T cells among CD26^neg/low/high^ subsets. **J** Representative plots of the frequency of Cutaneous Lymphocyte Antigen (CLA)^+^CD8^+^ T cells among CD26^neg/low/high^ cells. **K** The graphical illustrates the tendency of CLA^+^CD8^+^ T cells homing to skin following E/P Selectin binding on endothelial cells. **L** Cumulative data of the frequency of CLA^+^CD8^+^ T cells among CD26^neg/low/high^ cells. **M** Representative plots, and the frequency of migrated CD26^neg^, CD26^low^, and CD26^high^ CD8^+^ T cells at the baseline and after 18 h in response to (**N**) Fetal Bovine Serum (FBS-10%), (**O**) RANTES (10 nM), and (**P**) IL-18 (100 ng/ml). Error bars represent the median with an interquartile range. Each dot represents an individual human sample.

CD8^+^ T cell migratory ability is crucial for accessing tumor sites, and distinct homing receptors are involved in this process [74]. To better characterize the migratory capacity of CD26^+^CD8^+^ T cell subpopulations, we subjected them to further analysis for the expression of various homing receptors. We found that CD26^high^CD8^+^ T cells had significantly higher frequency and intensity of CCR5 expression than CD26^neg^ and CD26^low^CD8^+^ T cells in CLL patients (Fig. 5A–C, and Additional file 1: Fig. S4e, f). We made similar observations for the proportion/intensity of CCR6 (Fig. 5D–F, Additional file 1: Fig. S4g, h), and $\beta$ 7 Integrin in CD26^high^CD8^+^ T cells (Additional file 1: Fig. S4i, j). While CCR5 directs migration along RANTES, CCL3, and MIP-1 $\alpha /\beta$ to the secondary lymphoid organs and inflammation sites[75], CCR6 and integrin-$\beta$ 7 traffic T cells towards mucosal tissues such as the gut in response to CCL20 and MadCAM-1 [76, 77]. In contrast to CD26^high^, we found significantly a higher proportion of CCR7 expressing cells with greater intensity among CD26^low^CD8^+^ T cells (Fig. 5G–I, and Additional file 1: Fig. S4k, l), which implies these cells tend to home to lymph nodes in response to CCL19 [78]. Similar observations were made for the Common Lymphocyte Antigen (CLA), a skin homing-receptor via binding to E selectins [79], in CD26^low^CD8^+^ T cells (Fig. 5L, and Additional file 1: Fig. S4m, n). It is worth mentioning that we did not find any significant difference in the frequency/intensity of T cells expressing either CXCR3 or CXCR4 among CD26^neg^/CD26^low^/CD26^high^ CD8^+^ T cells in CLL patients (Additional file 1: Fig. S4o–r). Finally, we investigated the expression of CCR4 and found a higher advantage of CD26^+^ versus their CD26^neg^ CD8^+^ T cells counterparts for the expression of this skin homing receptor[75] (Additional file 1: Fig. S4s). To better delineate the migration capabilities of CD26^neg^/CD26^+^CD8^+^ T cells, we performed a trans-well migration assay on PBMCs from CLL patients. We observed that CD26^neg^ and CD26^high^ exhibited equally but significantly higher migratory capacity than their CD26^low^ counterparts toward a general chemoattractant (Fetal bovine serum 10%) when examined after 18 h (Fig. 5M, N). However, CD26^high^ cells displayed an enhanced migration ability towards RANTES and IL-18 compared to CD26^neg^/CD26^low^ CD8^+^ T cells, possibly due to higher expression of CCR5 and IL-18R $\alpha$ (Fig. 5M, O, P). We also measured the proportion of CD69-expressing cells among different CD26^±^ subsets. CD69, as an early activation marker, is reported to regulate the retention of T cells from the periphery into tissues to generate tissue-resident memory T cells [80]. Interestingly, we noted that CD26^high^ subset was significantly enriched with CD69 expressing cells compared to CD26^neg/low^ CD8^+^ T cells in CLL patients (Additional file 1: Fig. S5a, b). Collectively, our results suggest that CD26^high^CD8^+^ T cells have a greater migratory trait to peripheral organs such as the gut, mucosal surfaces, and inflamed tissues. In contrast, the CD26^low^ subset has a higher homing capacity to lymph nodes and skin.

### CD26^low^CD8^+^ T cells are long-lived compared to CD26^neg/high^CD8^+^ T cells

**Fig. 6:**
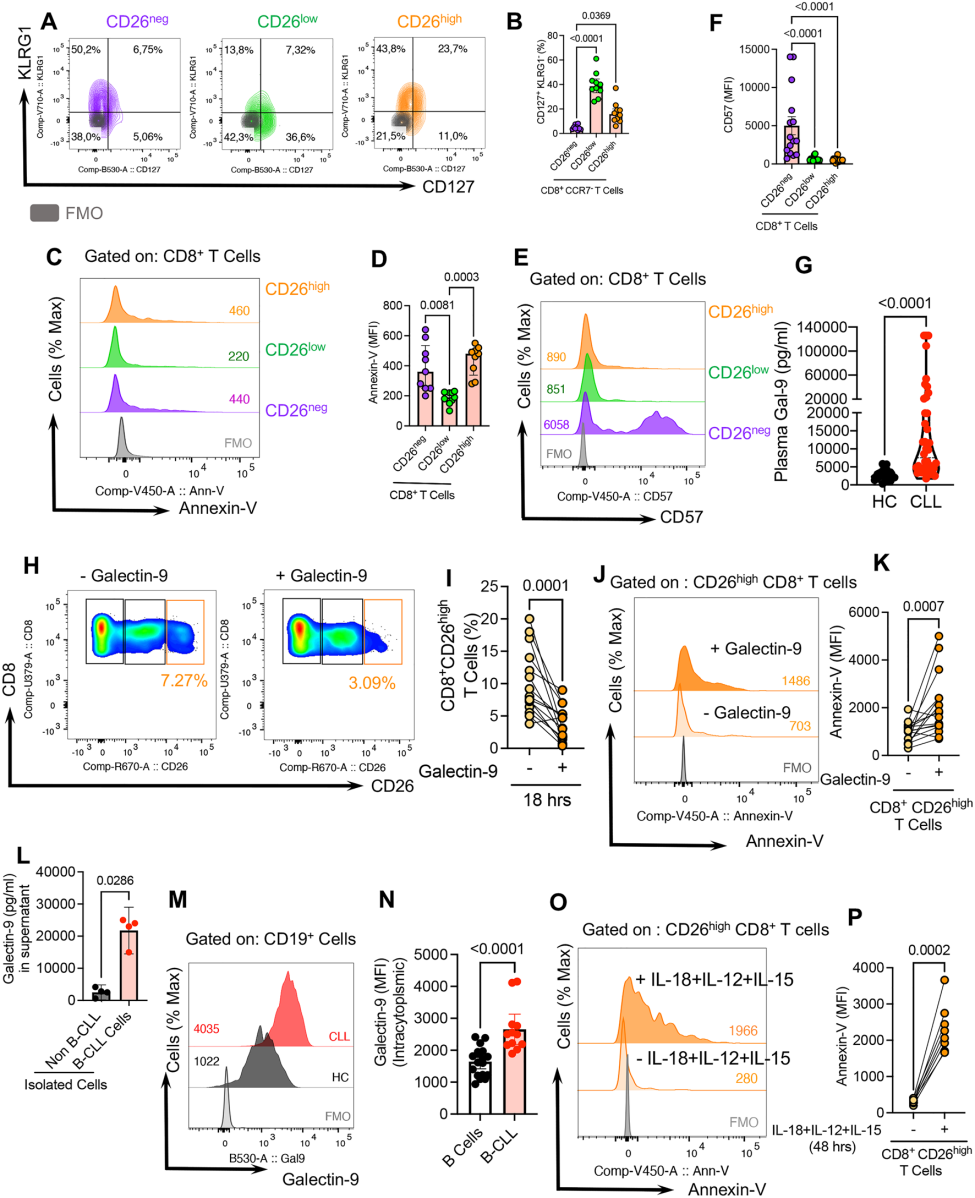
Gal-9 preferentially promotes CD26^high^ CD8^+^ T cells apoptosis in CLL. **A** Representative plots, and (**B**) cumulative data of the frequency of CD127^+^/KLRG1^−^ cells among CD26^neg/low/high^ subsets of CD8^+^ T cells. **C** Representative plots, and (**D**) cumulative data of the intensity of Annexin-V expression (MFI) among CD26^neg/low/high^ CD8^+^ T cells. **E** Representative plots, and (**F**) cumulative data of the MFI of CD57^+^ among CD26^neg/low/high^ CD8^+^ T cells. **G** Cumulative data of the plasma Gal-9 concentrations in HCs and CLL patients. **H** Representative plots, and (**I**) cumulative data of the frequency of CD26^high^CD8^+^ T cells following stimulation with anti-CD3/CD28 in the presence or absence of recombinant human Gal-9 (0.02 g$\mu$/ml). **J** Representative plots, and (**K**) cumulative data of the intensity of Annexin-V expression in CD26^high^ CD8^+^ T cells following stimulation with anti-CD3/CD28 in the presence or absence of Gal-9. **L** Cumulative data of Gal-9 concentration in the supernatants of isolated non-B cells versus malignant B cells (B-CLL) after 12 h culture. **M** Representative plots, and (**N**) cumulative data ofthe intracytoplasmic MFI of Gal-9 in B cells from HC versus B-CLLs. **O** Representative plots, and (**P**) cumulative data of the frequency of Annexin-V expressing CD8^+^ T cells in CD26^high^ cells at the baseline versus stimulation with a cytokine cocktail (IL-18 + IL-12 + IL-15) (100 ng/ml of each) for 18 h. Error bars represent the median with an interquartile range. Each dot represents an individual human sample

To evaluate the survival capacity of these three subpopulations of CD8^+^ T cells, we subjected them to KLRG1 and CD127 (IL-7R $\alpha )$ expression analysis because long-lived T cells express high levels of CD127 but low levels of KLRG1 [81]. Our studies show that CD26^low^CD8^+^ T cells have higher levels of CD127 but lower levels of KLRG1 expression in CLL patients (Fig. 6A, B). Given these observations, we next examined CD26^neg/low/high^ cells in terms of apoptosis. This apoptotic assay confirmed the longevity of CD26^low^CD8^+^ T cells as they showed lesser Annexin-V expression than their CD26^neg^ and CD26^high^ counterparts (Fig. 6C, D). In parallel, we measured the frequency of T stem cell memory (TSCM) [63] (CD45RA^+^CCR7^+^CD95^+^) in CD26^neg^, CD26^low^, and CD26^high^ subsets of CD8^+^ T cells. These analyses revealed that the CD26^low^ subset had a significantly higher proportion of TSCM cells compared to their CD26^neg/high^ counterparts, supporting a higher self-renewal propensity (Additional file 1: Fig. S5c).

As CD8^+^ T cells acquire terminal differentiation phenotype, they upregulate the expression of CD57 and/or CD16 along with GzmB and perforin. As a result, we observed a higher expression of CD57 and CD16 among CD26^neg^ CD8^+^ T cells (Fig. 6E, F, and Additional file 1: Fig. S5d). This is consistent with our previous observation of a higher GzmB and perforin expression in this subpopulation (Fig. 3A, B).

### CD26^high^CD8^+^ T cells are sensitive to Galectin-9 (Gal-9) induced apoptosis

To better investigate the mechanism associated with decreased CD26 frequency in CLL patients, we treated PBMCs from HCs with plasma (10%) obtained from CLL patients. After overnight culture, we found a significant reduction in the intensity of CD26 expression (Additional file 1: Fig. S5e, f). This observation suggested the presence of potential soluble mediator (s) in reducing CD26 expression in CLL patients. To identify the potential soluble factor, we performed multiplex ELISA and quantified 20 different cytokines/chemokines (Additional file 1: l Fig. S5g, h). We tested the effects of some of the most abundant cytokines on CD26 expression, however, these cytokines exhibited no effects or increased the intensity of CD26 expression in CD8 T cells (Additional file 1: Fig. S5i–n). Next, we measured the levels of TGF-β as a potential contributing factor in the attenuation of CD26, as reported in human breast cancer [82]. Although total TGF-β levels were the same in HCs and CLL patients, we noted a significant decrease in the plasma levels of active form of TGF-β in CLL patients (Additional file 1: Fig. S5o, p). In agreement with the other report in breast cancer, we observed a significant decline in CD26 expression upon treatment with TGF-β in vitro (Additional file 1: Fig. S5q). Notably, we discovered a moderate but inverse correlation between the plasma free TGF-β level with the percentages of CD26-expressing CD8^+^ T cells in CLL patients (Additional file 1: Fig. S5r). Although TGF-β may contribute to the reduction of CD26 levels, CLL patients had significantly lower levels of this cytokine in their plasma than HCs.

Considering the apoptotic effects of Gal-9 on highly activated CD8^+^ T cells [83, 84] and our previous studies [53] [85] we hypothesized that Gal-9 might contribute to the depletion of highly polyfunctional CD26^high^CD8^+^ T cells in CLL. Therefore, we found a significant elevation in the plasma Gal-9 levels in CLL patients versus HCs (Fig. 6G).

To test our hypothesis, we treated total CD8^+^ T cells from HCs with recombinant human Gal-9 (0.02 $\mu g/ml,$ physiologically relevant to the plasma levels) for 18 h. We observed a significant decrease in the frequency of the CD26^high^ subset (Fig. 6H, I), which was consistent with more robust apoptosis of CD26^high^CD8^+^ T cells Fig. 6J, K). Overall, we discovered that Gal-9 exhibited a more pronounced apoptotic effect on the CD26^high^ population than the CD26^low^ and CD26^neg^ subsets (Additional file 1: Fig. S5s). To identify the possible source of Gal-9, we cultured PBMCs from CLL and HCs overnight in vitro and subjected their culture supernatants to Gal-9 quantification. This study revealed a significantly higher Gal-9 shedding in PBMCs from CLL patients (Additional file 1: Fig. S5t). Our further analysis confirmed B-CLLs as the major source of Gal-9 when compared to their non-B-CLL counterparts (Fig. 6L). Moreover, we found significantly higher levels of intracytoplasmic Gal-9 in B-CLL compared to healthy B cells (Fig. 66M, N). These results suggest that malignant B cells are a significant source of increased Gal-9 in CLL. Also, due to the elevated levels of plasma IL-18, IL-12, and IL-15 in CLL patients (Additional file 1: Fig. S5u–w), we assessed the potential effects of these cytokines on CD26 expression. We found that this cytokine cocktail, at physiological concentration detected in the plasma, significantly enhanced apoptosis of CD26^high^CD8^+^ T cells (Fig. 6O, P, and Additional file 1: Fig. S6a). Overall, these observations suggest that B-CLL cells as a major source of elevated Gal-9 in CLL plasma could contribute to the depletion of CD26^high^CD8^+^ T cells. Alternatively, IL-18 + IL-12 + IL-15 may promote apoptosis of CD26^high^CD8^+^ T cells in CLL. However, these cytokines individually do not impact the expression of CD26.

### CD26^+^ T cells in mice have a different phenotype than their counterparts in humans

To investigate further the role of CD26 cells in an animal model, we measured the frequency of CD26 in CD8^+^ T cells of BALB/c mice. Surprisingly, we found that nearly 100% of CD8^+^ T cells in mice regardless of their niche expressed CD26 (e.g. thymus, blood, and spleen) (Additional file 1: Fig. S6b, c). In addition, we did not see CD26^high/low^ subpopulations in CD8^+^ T cells of mice, therefore, the CD26 expression pattern is completely different in mice than humans. More importantly, our observations show that CD26^high^CD8^+^ T cells are enriched with MAIT cells in humans (Fig. 2); however, mice MAIT cells have a CD44^high^CD62^LOW^ phenotype [86]. These observations demonstrate that CD26-expressing cells have a different phenotype in humans than mice.

## Discussion

CD8^+^ T cells become dysfunctional/exhausted during chronic conditions such as cancer [85, 87]. Clinical approaches such as immune checkpoint blockers and adoptive immune cell therapies have shown promising outcomes in different cancer types. One advantage of adoptive cell therapy is identifying and infusing selected polyfunctional CD8^+^ T cells with enhanced antitumor properties into cancer patients. Our study provides a novel insight into the immunological properties of CD26^+^CD8^+^ T cells in CLL patients. We observed significant decline of polyfunctional CD26^+^CD8^+^ T cells in CLL patients.

First, we stratified CD26^+^CD8^+^ T cells into CD26^low^/CD26^high^ and observed that the percentage and total number of these cells were declined in CLL patients. We discovered that CD26^high^CD8^+^ T cells were mainly transitional and effector memory cells, as reported elsewhere [28]. However, the CD26^low^ subset was highly enriched with naïve, stem cell and central memory CD8^+^ T cells. Our finding that both CD26^high^ and CD26^low^ were augmented with CD27^+^ T cells suggests that such cells may have a selective advantage compared to CD26^neg^CD8^+^ T cells. The increased proliferative capacity and IL-2 production by CD26^high^/CD26^low^ cells likely reflect a costimulatory signal from CD27 vis NF-κB activation [88]. The presence of such signal from CD27 may contribute to the enhanced survival and persistence of antigen-specific CD8^+^ T cells to protect the host from malignancy, as reported in HIV-infected individuals [89]. Moreover, we found that CD26^high^CD8^+^ T cells displayed a Tc1/Tc17 phenotype. This was illustrated by the abundance of CXCR3/CCR6/CCR4 expressing cells in this subpopulation [72]. Notably, we noted that CD26^high^CD8^+^ T cells had a prominent propensity to exhibit cytotoxic properties by high expression of GzmB, perforin, and IFN $-\gamma$ upon stimulation with a cytokine cocktail (IL-12 + IL-18 + IL-15) or TCR, as reported for MAIT cells [90]. Of note, CD26^+^CD8^+^ T cells exhibited a more robust response to the cytokine cocktail than TCR-induced stimulation. Furthermore, they displayed a greater IL-2 expression capacity. This intrinsic IL-2 production capacity of CD26^high^CD8^+^ T cells may enable them to have a stemness-like feature as reported in chronic viral infection[91]. In addition, elevated GzmK contents in CD26^high^CD8^+^ T cells in a quiescent state, along with stimulation-induced upregulation of GzmB, further support their polyfunctionality [92].

Moreover, we characterized CD26^+^CD8^+^ T cells based on the defined surrogate markers for MAIT cells (CD161^high^ TV $\alpha$ 7.2^+^) [65]. Although CD26^high^CD8^+^ T cells were enriched with MAIT-like cells, they display a heterogenous subset of CD8^+^ T cells in CLL patients. This is consistent with another reports that MAIT-like surrogate markers can be affected by the disease status [65] and are not definite markers for MAIT cell identification. To overcome this issue, the implication of MR-1 tetramers as a confirmatory approach for the identification of MAIT cells within CD26^high^CD8^+^ T cells has been suggested [65]. Also, we noted a higher ROR $\gamma \delta$ expression in CD26^high^ CD8^+^ T cells in favor of a Tc17 or MAIT17 phenotype [72]. The plasticity of Tc17 cells and their either protective or pathogenic role in the context of cancer has been the subject of controversy [93]. Therefore, further studies are required to appreciate better the role of CD26^high^CD8^+^ T cells in CLL patients and other solid cancers.

Moreover, we found that CD26^high^CD8^+^ T cells express elevated levels of IL-18R $\alpha$, which enables them to respond to cytokine-induced stimulation (e.g. IL-18) and migrate toward the inflammation site as reported for MAIT cells in bacterial infection [94].

In addition, we found it intriguing that CD26^high^CD8^+^ T cells express chemokine receptors such as CCR5, CCR6, integrin-$\beta 7,$ and CD69, which possibly promotes the trafficking of CD26^high^CD8^+^ T cells into inflamed tissues (mucosal sites) and tumors. Collectively, these capabilities signify the multifunctional and plasticity of CD26^high^ cells to protect against bacterial infections and tumors. Based on our observations, we posit that CD26^high^CD8^+^ T cell deficiency might be one potential explanation for the increased susceptibility to recurrent bacterial infections and tumor progression in CLL patients. Therefore, CD26^high^CD8^+^T cells may employ key mechanisms such as polyfunctionality, migration, and stemness to survive and destroy cancer cells, as reported for MAIT cells [95].

In contrast to CD26^high^T cells, we discovered that CD26^low^CD8^+^ T cells mainly were naïve, stem cells memory, and central memory, but their frequency gradually decreased as they differentiated into transitional memory, effector memory, and effector T cells. Although CD26^high^ and CD26^low^CD8^+^ T cells displayed some similar characteristics, they were distinct in many aspects. For instance, CD26^low^CD8^+^ T cells acquired cytotoxic properties upon TCR and cytokine-triggered stimulation. In addition, we observed that CD26^low^CD8^+^ T cells were long-lived memory T cells (CD127^+^ KLRG1^−^) with higher proliferative capabilities and more stemness-cell- like features, as reported elsewhere [96]. These properties make CD26^low^CD8^+^ T cells a potential reservoir of long-lived memory cells with crucial roles in immune homeostasis and response to tumor cells.

To gain a better insight into different CD8^+^ T cell subsets, we also studied CD26^neg^CD8^+^ T cells in CLL and HCs. These studies revealed that CD26^neg^CD8^+^ T cells mimicked antigen-experienced T cells. The main crowd of CD25^neg^CD8^+^ T cells closely resembled transitional, effector memory, and effector T cells. We found that CD26^neg^ T cells enriched with CXCR3/CCR4 expression and displayed a Tc1/Tc2 profile. Furthermore, these cells contained high levels of GzmB and perforin content in the absence of reactivation, and TCR or cytokine-mediated stimulation did not impact their cytolytic molecules expression capabilities in vitro. This suggests that CD26^neg^CD8^+^ T cells are at their maximum functional potentials with minimal plasticity. In addition to cytolytic properties (high GzmB, perforin, and CD107 expression), CD26^neg^ T cells had elevated levels of CD57 and CD16, which fulfills the criteria of terminal effector T cells [97, 98]. Moreover, we found that CD26^neg^ T cells express elevated levels of co-inhibitory receptors (e.g., CD160, TIGIT, 2B4, CD39) but lower levels of co-stimulatory receptors (e.g., CD28, CD27). These observations beg the question of whether CD26^neg^ T cell display an exhausted phenotype in CLL patients. This hypothesis was supported by their lower cytokine expression (e.g. IFN-γ and IL-2), proliferative capacity, and minimal responsiveness to in vitro stimulation.

We found it intriguing that CD26^neg^ T cells substantially had lower CD28 expression than their CD26^low/high^ siblings. Although we were unable to delineate the underlying mechanism, gradual downregulation of CD28 expression in T cells in response to chronic antigenic stimulation and aging has been reported [99], as we observed in our cohort (Additional file 1: Fig. S6d). Also, the elevated expression of CD57 levels in the CD26^neg^ T cell subpopulation supports the concept of chronic antigen-dependent differentiation and proliferation [100].

Although the role of CD26^+^CD8^+^ T cells in cancer models has not been investigated, polyfunctional CD4^+^CD26^high^ T cells display markers of stemness/migration and elicit anti-tumor activity in different malignancies [26]. On the contrary, the expression of CD26^+^ on cancer cells is linked to stemness, invasiveness, and increased metastatic capability [101]. Moreover, the enzymatic activity of CD26 in cancer models has not been fully understood. For example, one group reported that CD26 inhibition was associated with improved anti-tumor immunity [102]. However, another group showed that CD26 inhibition promotes tumor progression/metastasis [103]. As such, further investigation on the immunological role of CD26 beyond T cells in cancer models is needed. Such studies will enable us to determine whether genetic or therapeutic manipulation of CD26 expression can promote anti-tumor immunity.

**Fig. 7:**
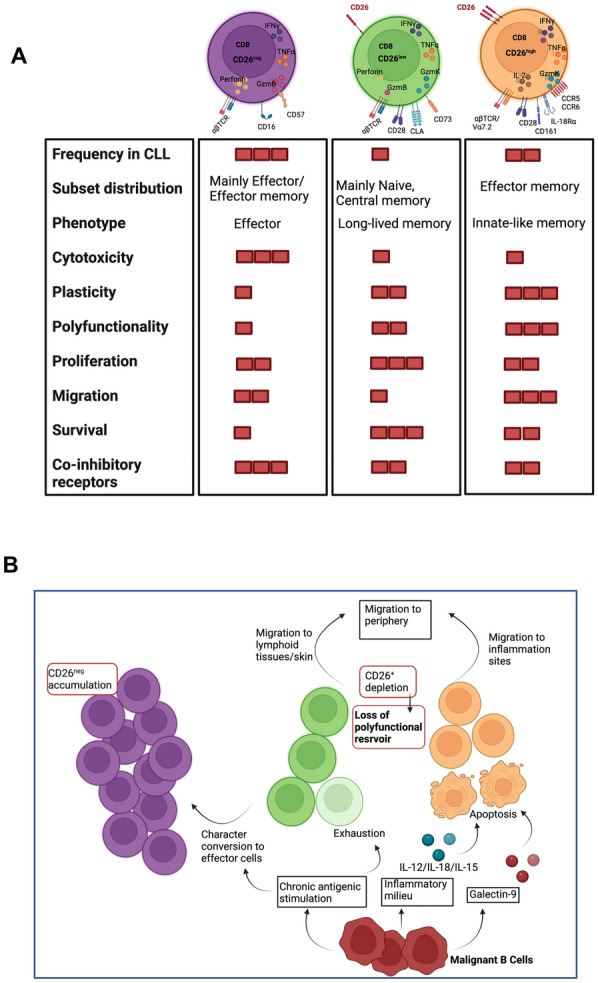
Visual summary. **A** CD26 expression defines three distinct populations of CD8^+^ T cells in CLL with discrete properties. The table summarizes different properties of CD26^neg^, CD26^low^, and CD26^high^ CD8^+^ T cells ranked as high (∎∎∎) moderate (∎∎), and low (∎) **B** The illustration depicts the proposed mechanism of decreased frequency of CD26^low^ and CD26^high^ subsets and the expansion of CD26^neg^ CD8^+^ T cells in CLL. Migration towards inflamed tissues, elimination by apoptosis (Gal-9, Inflammatory cytokines), change of character/exhaustion due to chronic antigenic stimulation in CLL are proposed as potential mechanisms leading to the depletion of CD26^+^CD8^+^ in CLL

This is the first study, to our knowledge, that characterizes human CD8^+^ T cell subsets by CD26 expression and analyses their effector functions in CLL patients versus HCs. Specifically, our major findings are fourfold. The major results of our findings are summarized in the table shown in Fig. 7A. Firstly, we found that CD8^+^ T cells expressing either low or high levels of CD26 were decreased in CLL patients. Secondly, our observations revealed that CD26^low/high^ T cells were not terminally differentiated compared to CD26^neg^ T cells. The CD26^high^ T cell subset had transitional/effector memory and CD26^low^ naïve and stem cell/central memory phenotype. Thirdly, the higher migration capacity of CD26^+^ T cells may support their trafficking to lymph nodes and inflamed organs or the tumor microenvironment (TME). Whether this migratory capacity explains their deficiency in blood circulation needs to be determined. Fourthly, our finding that CD26^high^/CD26^low^ CD8^+^ T cells are polyfunctional and exhibit greater migratory capacity, stemness, longevity, and proliferation capability make them a potential candidate for adoptive T cell transfer or CAR T cell therapy in CLL. Moreover, we found elevated levels of Gal-9 in the plasma of CLL patients. Considering particular apoptotic properties of Gal-9 on CD8^+^ T cells, we discovered that CD26^high^CD8^+^ T cells were susceptible to apoptosis following exposure to Gal-9 and IL18 + IL12 + IL-15 in vitro. Therefore, the inflammatory milieu of CLL with the elevated levels of IL-18, IL-12, IL-15, and Gal-9 that are released from CLL cells might explain a mechanism that results in the reduction of CD26^high^CD8^+^ T cells pool in CLL (Fig. 7B). More importantly, Gal-9 is strongly associated with the elevation of pro-inflammatory cytokines/chemokines [54]. As such, Gal-9 might be involved in the inflammatory cascade and indirectly compromises anti-tumor immunity by depleting polyfunctional CD26^+^CD8^+^ T cells in CLL. Whether targeting Gal-9 could prevent the elimination of CD26^+^ T cells in CLL merits further investigations.

We are aware of multiple study limitations. The age factor might impact CD26^+^ T cell frequency [104]; however, this was not the case in our cohort.

Notably, a decline in CD26^+^CD8^+^ T cells has been reported in chronic viral infections such as HIV, CMV, and EBV [33]. Therefore, there is a possibility that CD26^high^CD8^+^ T cells, due to their enhanced trafficking abilities, are attracted to the gut and inflamed tissues whereas CD26^low^ T cells are more likely to home to skin and lymph nodes. From another perspective, decreased CD26^+^CD8^+^ T cells might be considered as a potential predisposing mechanism for increased infection rates in CLL patients. Another limitation of our study was a small/single-centered cohort. Therefore, we strongly recommend performing similar studies in larger multiple-centered cohorts and different hematological malignancies and solid tumors to appreciate better the role of CD26^+^CD8^+^ T cells in cancer. Although we know that T cells in the blood circulation differ strongly from lymph node-derived T cells in CLL, performing such an invasive procedure is uncommon in CLL patients.

Finally, we could not obtain a larger blood volume for conducting more in-depth analysis such as RNAseq or single-cell sequencing on different CD26^+^ T cell subsets in CLL patients. Such studies will enable us to better characterize this T cell subset for therapeutic interventions.

## Conclusion

In conclusion, our findings demonstrate the depletion of highly polyfunctional CD26^+^CD8^+^ T in CLL. These cells exhibit greater migratory capacity, stemness, longevity, and proliferation capability, which make them a potential candidate for adoptive T cell transfer or CAR T cell therapy in CLL. Considering the role of CD26: ADA in converting adenosine to inosine, CD26^+^ T cells could bypass the immune suppressive effects of adenosine in the TME and periphery. Also, our results indicate the involvement of Gal-9 in the inflammatory cascade and indirectly compromises anti-tumor immunity by depleting polyfunctional CD26^high^CD8^+^ T cells in CLL. Therefore, targeting Gal-9 to persevere polyfunctional T cells in CLL merits further investigations and should be considered.

## Supplementary Information

**Additional file 1: Figure. S1.** **a** Representative flow plot of the purity of isolated CD3^+^ T cells, (**b**) CD3^+^CCR7^-^ cells, and (**c**) CD19^+^ B cells. **d** Representative flow plots of the gating strategy for CD26 staining in CD8^+^ T cells. **e** Cumulative data showing the number of CD26^low^ and CD26^high^ CD8^+^ T cells as normalized in 100,000 CD8^+^ T cells in HC and CLL patients. **f** Cumulative data comparing the Mean Fluorescence Intensity (MFI) of CD26 in CD26^low^ and CD26^high^ CD8^+^ T cells. **g** Cumulative data comparing the frequency of CD26^+^CD8^+^ T cells in female versus male CLL patients. **h** Correlation between the age of CLL patients and the frequency of CD26^+^CD8^+^ T cells. **i** Cumulative data comparing the frequency of CD26^+^ and, (**j**) CD26^low^, CD26^high^ CD8^+^ T cells in treated versus non-treated CLL. **k** Cumulative data are comparing the proportion of CD26^+^CD8^+^ T cell in three clinical stages of CLL (Low/Intermediate/high) based on the Rai staging system. **l** Correlation between CD26^+^CD8^+^ T cell frequency and lymphocyte counts (x10^3^/$\mu$l) in CLL. **m** Representative flow plots, and (**n**) cumulative data of the frequency of CD26 among CD3^-^ and CD3^+^ T cells in HC and CLL. (**o**) Cumulative data showing the frequency of CD26^+^CD4^+^ T cells, and (**p**) CD56^+^NK cells in HC versus CLL. **q** Cumulative data showing the MFI of CD26 in B cells from HCs and malignant B cells (B-CLL). **r** Cumulative data of the concentrations of soluble CD26 (ng/ml) in the plasma of HC and CLL. **s** Cumulative data of the frequency of CD26^+^, and (**t**) CD26^low^, CD26^high^ CD8^+^ T cell in the peripheral blood versus bone marrow of CLL. **u** Cumulative data of CD26 mRNA expression in CD8^+^ T cells of HCs vs. CLL (n=15). Error bars represent the median with an interquartile range. Each dot represents an individual human sample. **Figure. S2.** **a** Representative plots, and (**b**) cumulative data of the frequency of CD8^+^ T cell subsets (e.g. naïve, stem cell memory, central memory, transitional memory, effector memory, and effectors). **c** Representative flow plots, and (**d**) cumulative data showing the frequency of CD27 expressing cells among CD26^neg/low/high^ effector memory subsets of CD8^+^ T cells in CLL. **e** Representative plots, and (**f**) Cumulative data of the frequency of TV$\alpha$7.2^+^CD161^high^ in CD26^high^ CD8^+^ T cells in HC. **g** Cumulative data of the frequency of TV$\alpha$7.2^+^ CD161^high^ in CD26^high^CD8^+^ T cells in HC versus CLL. **h** Cumulative data of the frequency of CD160^+^, (**i**) 2B4+, (j) PD-1^+^, (**k**) TIGIT^+^, (**l**) ICOS^+,^ (**m**) CD28^+^ , (**n**) CD27^+^, (**o**) CD39^+^ , and (p) CD73^+^ among CD26^neg^, CD26^low^, and CD26^high^ CD8^+^ T cells in HC. **q** Representative plots of the co-expression of CD26 and CD73 in different CD8^+^ T cell subsets (naïve, central memory, effector memory, and effector) in CLL. **r** The pie charts show the pattern of CD26 and CD73 co-expression in different CD8^+^ T cell subsets in CLL. **s** Cumulative data of the frequency of CD26^+^CD73^+^ versus CD26^+^CD73^-^ in CD8^+^ T cells of HC versus CLL. **t** Cumulative data of the frequency of co-inhibitory/co-stimulatory receptors in CD26^high^CD8^+^ T cell subset in HC and CLL. Error bars represent the median with an interquartile range. Each dot represents an individual human sample. **Figure. S3.** **a** Cumulative data of the frequency of co-inhibitory/co-stimulatory receptors in CD26^neg^ CD8^+^ T cell subset in HC and CLL. **b** Cumulative data of the frequency of GzmB^+^, (**c**) Perforin^+^, and (**d**) GzmB^+^Perforin^+^ among CD26^neg^, CD26^low^, and CD26^high^ CD8^+^CCR7^-^ T cells in CLL. **e** Cumulative data of the frequency of GzmB^+^, (f) Perforin^+^, and (**g**) GzmB^+^Perforin^+^ CD8^+^ T cells among CD26^neg^, CD26^low^, and CD26^high^ subsets in HC. **h** Representative plots, and (**i**) cumulative data of the frequency of GzmB and perforin expressing cells among CD26^neg^, CD26^low^, and CD26^high^ CD8^+^ T cells in CLL either unstimulated (black color) or stimulated for following 5 h with anti-CD3/CD28 (3$\mu$g/ml, 1$\mu$g/ml). **j** Cumulative data of the frequency of TNF-$\alpha$^+^, (**k**) IFN-$\gamma$^+^, and (**l**) TNF-$\alpha$^+^IFN-$\gamma$^+^ cells among CD26^neg^, CD26^low^, and CD26^high^ CD8^+^ T cells in CLL. **m** Cumulative data of the frequency of TNF-$\alpha$^+^, (**n**) IFN-$\gamma$^+^, and (**o**) TNF-$\alpha$^+^IFN-$\gamma$^+^ among CD26^neg^, CD26^low^, and CD26^high^ CD8^+^ T cells in HC. **p** Representative plots, and cumulative data of the frequency of (**q**) CCR4^+^CCR6^+^ cells among CD26^neg^, CD26^low^, and CD26^high^ CD8^+^ T cells in CLL, considered as Tc17 cells. **r** Representative plots, and (s) cumulative data of the frequency of CXCR3^+^CCR6^+^ expressing cells among CD26^neg^, CD26^low^, and CD26^high^ CD8^+^ T cells in CLL, considered as Tc1/Tc17 cells. **t** Cumulative data of the frequency of CXCR3^+^CCR6^-^ among CD26^neg^, CD26^low^, and CD26^high^ CD8^+^ T cells in CLL, considered as Tc1 cells. **u** Cumulative data showing the frequency of CCR4^+^CCR6^-^ among CD26^neg^, CD26^low^, and CD26^high^ CD8^+^ T cells in CLL, considered as Tc2 cells. Error bars represent the median with an interquartile range. Each dot represents an individual human sample. Florescence minus one (FMO). **Figure. S4.** **a** Cumulative data of the expression of T-bet in different CD8^+^ T cells subsets in CLL. **b** Cumulative data of the expression of FOXP3 in different CD8^+^ T cells subsets in CLL. **c** Representative plots, and (**d**) cumulative data showing the intensity (MFI) of TOX expression among CD26^neg^, CD26^low^, and CD26^high^ CD8^+^ T cells in CLL. **e** Representative plots, and (**f**) cumulative data showing the intensity (MFI) of CCR5 expression among CD26^neg^, CD26^low^, and CD26^high^ CD8^+^ T cells in CLL. **g** Representative plots, and (**h**) cumulative data showing the intensity of CCR6 expression in CD26^neg^, CD26^low^, and CD26^high^ CD8^+^ T cells in CLL. **i** Representative plots, and (**j**) cumulative data showing the intensity of Integrin-$\beta 7$ expression in CD26^neg^, CD26^low^, and CD26^high^ CD8^+^ T cells in CLL. **k** Representative plots, and (**l**) cumulative data of the intensity of CCR7 expression in CD26^neg^, CD26^low^, and CD26^high^ CD8^+^ T cells in CLL. **m** Representative plots, and (**n**) cumulative data of the intensity of CLA (Cutaneous Lymphocyte Antigen) expression in CD26^neg^, CD26^low^, and CD26^high^ CD8^+^ T cells in CLL. **o** Cumulative data of the frequency, and (**p**) the intensity of CXCR3^+^ expression in CD26^neg^, CD26^low^, and CD26^high^ CD8^+^ T cells in CLL. **q** Cumulative data of the frequency, and (r) the intensity of CXCR4^+^ expression in CD26^neg^, CD26^low^, and CD26^high^ CD8^+^ T cells in CLL. **s** Cumulative data of the intensity of CCR4 expression in CD26^neg^, CD26^low^, and CD26^high^ CD8^+^ T cells in CLL. Error bars represent the median with an interquartile range. Each dot represents an individual human sample. **Figure. S5.** **a** Representative plots, and (**b**) cumulative data of the frequency of CD69 expressing cells among CD26^neg^, CD26^low^, and CD26^high^ CD8^+^ T cells in CLL. **c** Cumulative data showing the frequency of TSCM (T Stem Cell Memory: CCR7^+^ CD45RA^+^CD95^+^) among CD26^neg^, CD26^low^, and CD26^high^ CD8^+^ T cells in CLL. **d** Cumulative data showing the frequency of CD16^+^ in CD26^neg^, CD26^low^, and CD26^high^ CD8^+^ T cells in CLL. **e** Representative histogram plots, and (**f**) cumulative data of CD26 expression in CD8^+^ T cells upon culture with 10% plasma. (**g**, **h**) showing detected plasma concentrations of different cytokines and chemokines in CLL versus HCs. Cumulative data showing the intensity of CD26 in the presence/absence of (**i**) TNF-α, (**j**) IL-16, (**k**) IFN-γ, (**l**) IL-6, (**m**) IL-10, and (n) IFN-α. **o** Detected total plasma TGF-β and (**p**) free TGF-β in CLL versus HCs. **q** Cumulative data showing the intensity of CD26 expression in CD8^+^ T cells in the presence/absence of free TGF-β. **r** The correlation of plasma free TGF-β with the frequency of CD26^+^CD8^+^ T cells in CLL patients. **s** Cumulative data showing the intensity of Annexin-V expression in CD26^neg^, CD26^low^, and CD26^high^ CD8^+^ T cells from CLL patients following treatment with recombinant human Gal-9 (0.02 $\mu$g/ml) in-vitro. **t** Concentrations of Gal-9 (pg/ml) in supernatants of PBMCs (1× 10^6^ cells/well) from HC and CLL were collected 18 h post culture. **u** Concentrations of IL-18 (pg/ml) (**v**) IL-12/IL-23p40 (pg/ml), and (**w**) IL-15 (pg/ml) in the plasma of HC versus CLL. Error bars represent the median with an interquartile range. Each dot represents an individual human sample. **Figure. S6.** **a** Cumulative data showing the intensity of Annexin-V expression in CD26^neg^, CD26^low^, and CD26^high^ CD8^+^ T cells from CLL patients following 8 h of culture in the presence of a cytokine cocktail (IL-18+IL-12+IL-15 (100 ng/ml of each)). **b** Representative flow cytometry plots, and (**c**) cumulative data of CD26-expressing cells among CD8^+^ T cells in the thymus, blood, and spleen of BALB/c mice. **d** Cumulative data showing the frequency of CD28^+^CD8^+^ T cells in HC versus CLL. Error bars represent the median with an interquartile range. Each dot represents an individual human sample or a mouse. Florescence minus one (FMO).

**Additional file 2: Table S1.** Demographic and clinical information.

## Data Availability

The datasets used or analyzed during this study are included within the main article and Additional file materials.

## Abbreviations

ICOS: The inducible T cell co-stimulator
TIGIT: T cell immunoreceptor with Ig and ITIM domains
PD-1: Programmed cell death protein 1
IGHV: The immunoglobulin heavy chain gene
TOX: Thymocyte selection-associated HMG box
