# Supplementary figures and images for: Depletion of polyfunctional CD26highCD8+ T cells repertoire in chronic lymphocytic leukemia
Source: Exp Hematol Oncol. 2023 Jan 27;12:13. doi: 10.1186/s40164-023-00375-5 (PMC9881277; doi:10.1186/s40164-023-00375-5)

Additional file 1: fig S1

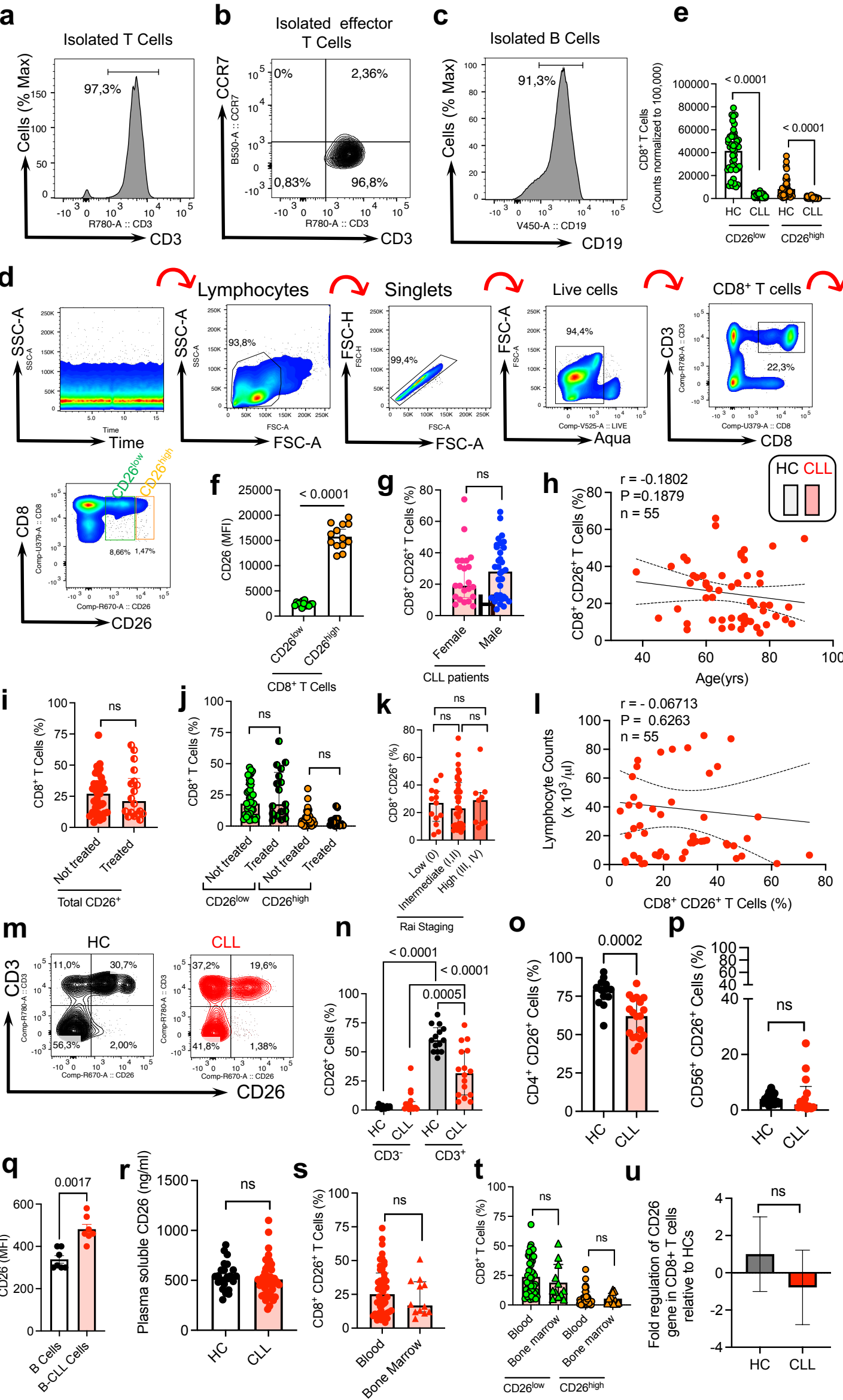

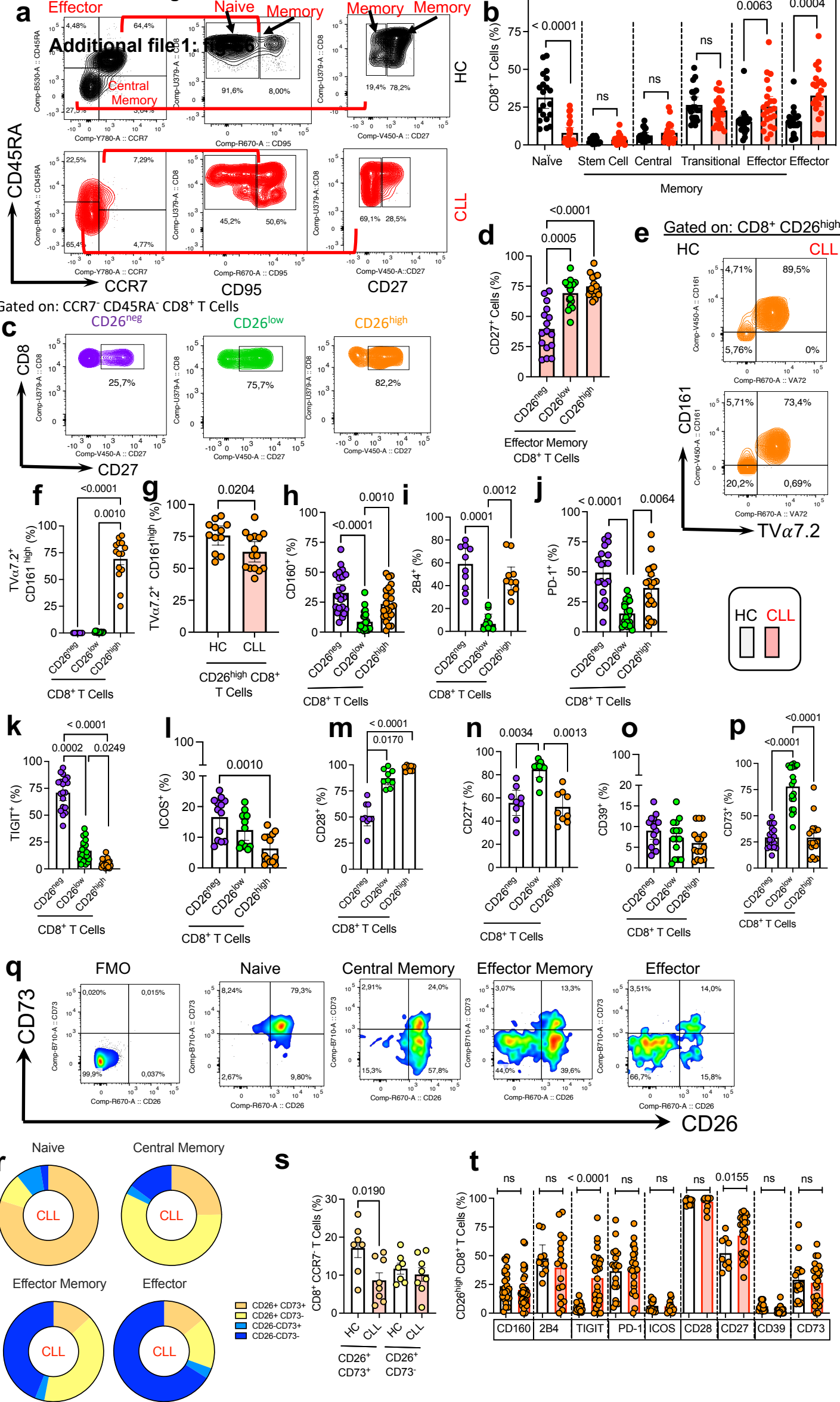

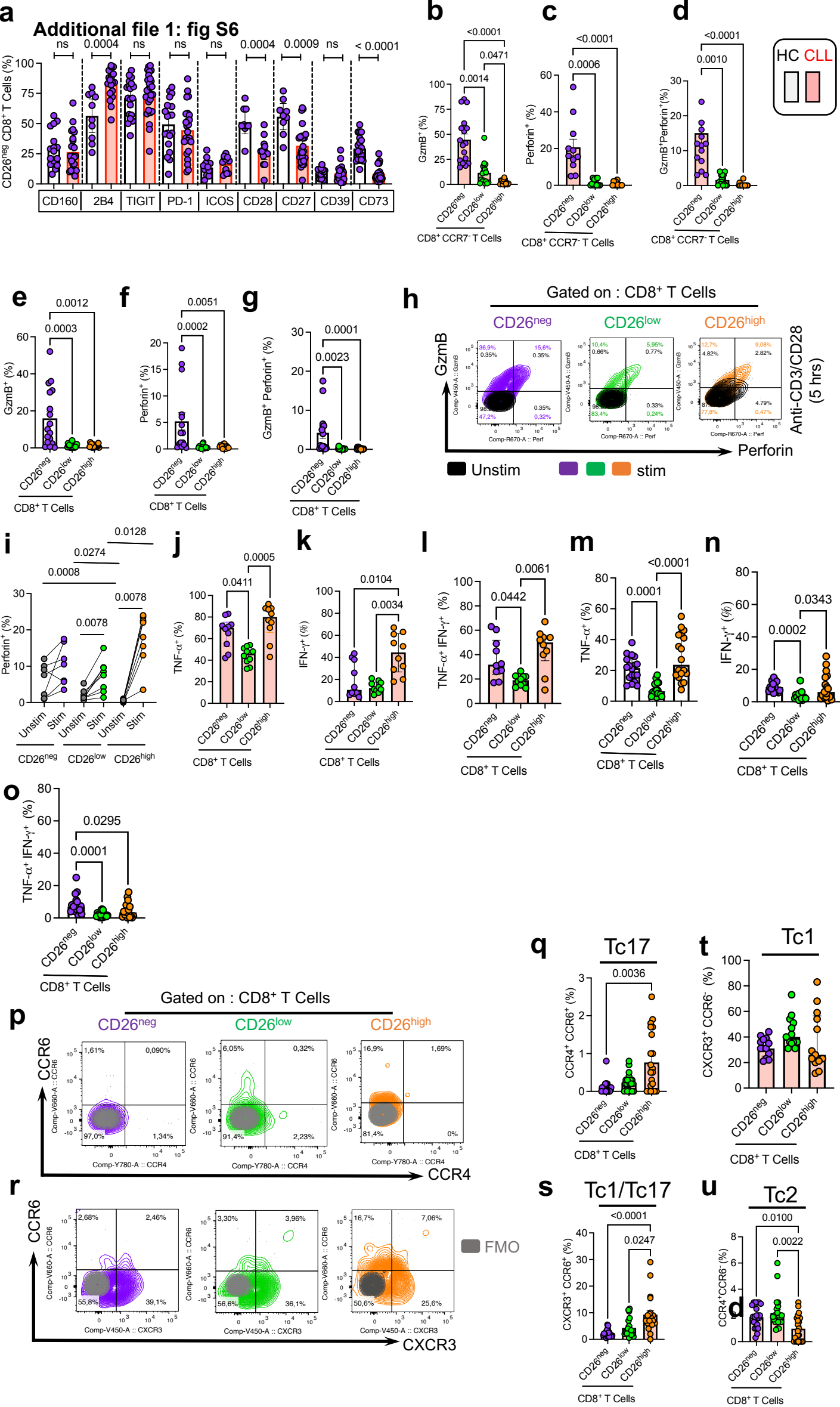

Additional file 1: fig S4

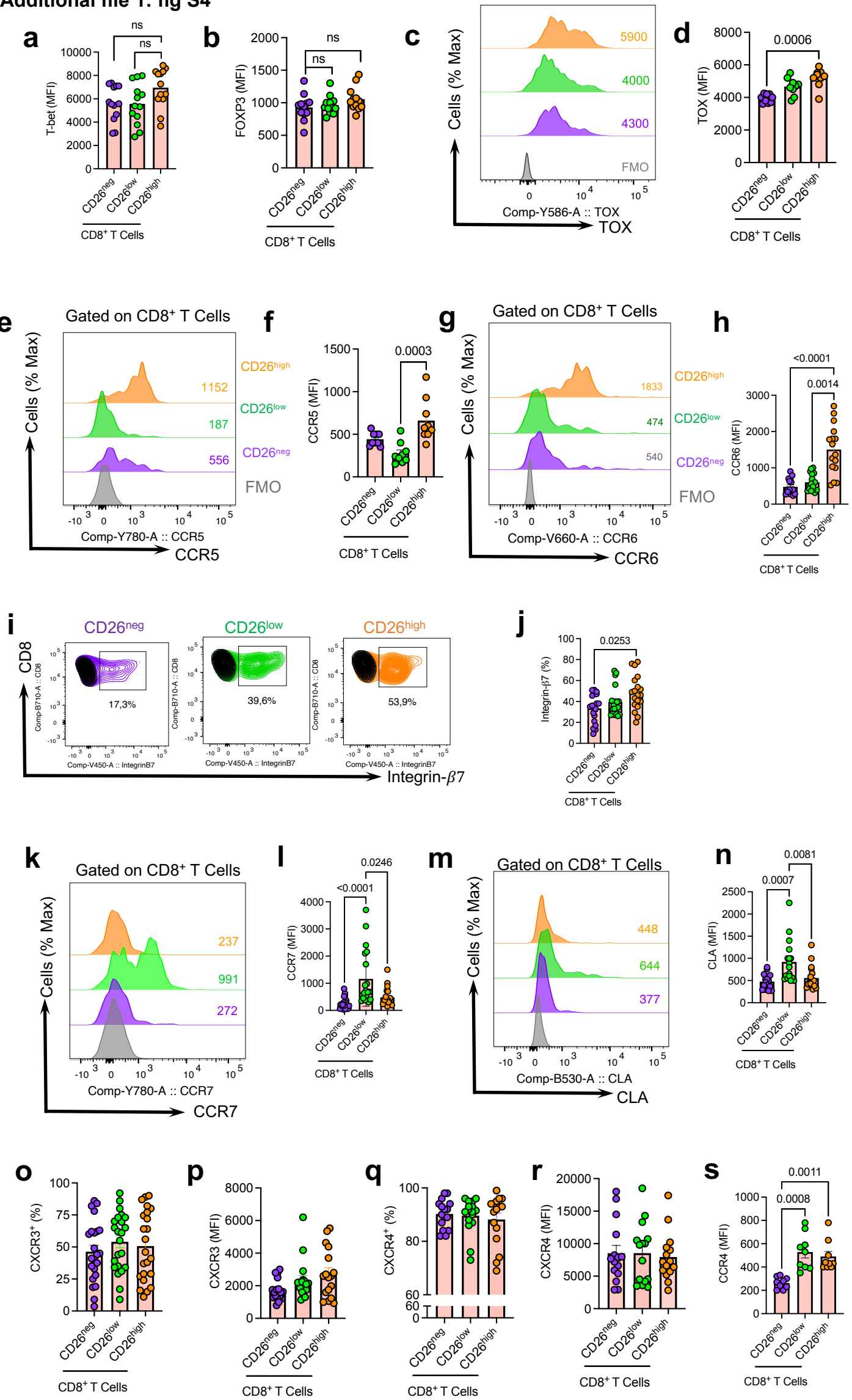

Additional file 1: fig S5

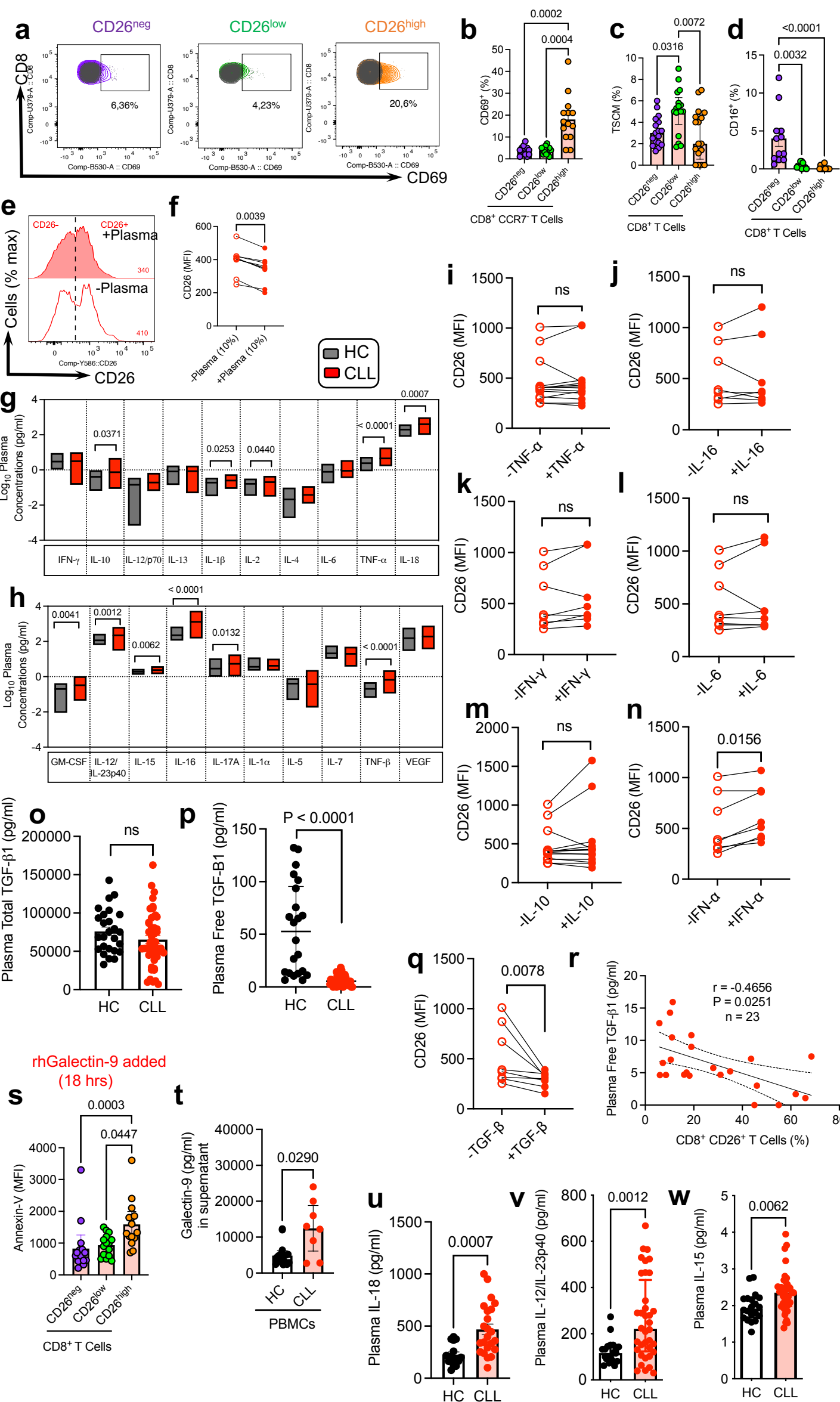

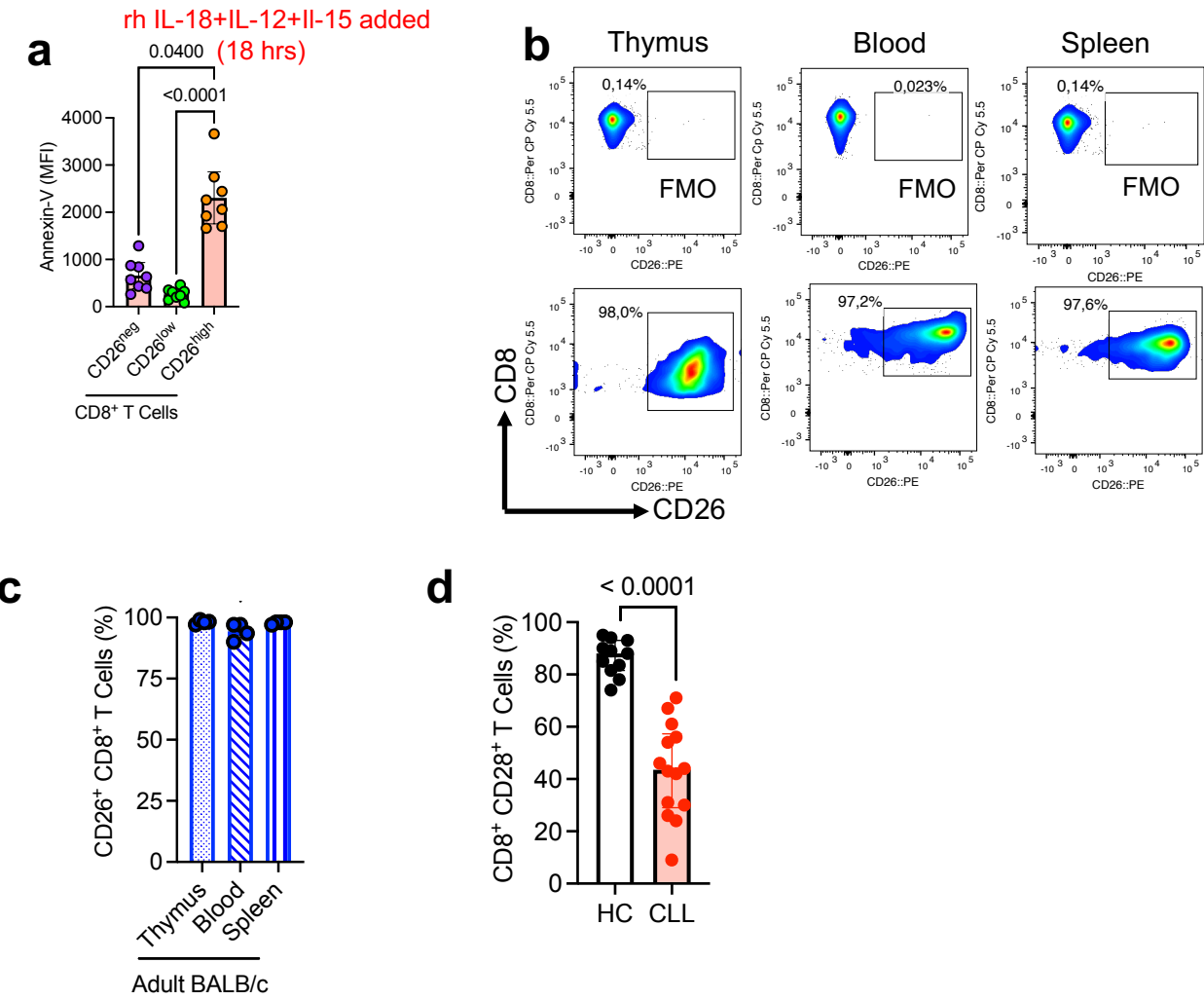

Supplement: Supplementary file 1 — Additional file 1: Figure. S1. a Representative flow plot of the purity of isolated CD3+ T cells, (b) CD3+CCR7- cells, and (c) CD19+ B cells. d Representative flow plots of the gating strategy for CD26 staining in CD8+ T cells. e Cumulative data showing the number of CD26low and CD26high CD8+ T cells as normalized in 100,000 CD8+ T cells in HC and CLL patients. f Cumulative data comparing the Mean Fluorescence Intensity (MFI) of CD26 in CD26low and CD26high CD8+ T cells. g Cumulative data comparing the frequency of CD26+CD8+ T cells in female versus male CLL patients. h Correlation between the age of CLL patients and the frequency of CD26+CD8+ T cells. i Cumulative data comparing the frequency of CD26+ and, (j) CD26low, CD26high CD8+ T cells in treated versus non-treated CLL. k Cumulative data are comparing the proportion of CD26+CD8+ T cell in three clinical stages of CLL (Low/Intermediate/high) based on the Rai staging system. l Correlation between CD26+CD8+ T cell frequency and lymphocyte counts (x103/$\mu$μl) in CLL. m Representative flow plots, and (n) cumulative data of the frequency of CD26 among CD3- and CD3+ T cells in HC and CLL. (o) Cumulative data showing the frequency of CD26+CD4+ T cells, and (p) CD56+NK cells in HC versus CLL. q Cumulative data showing the MFI of CD26 in B cells from HCs and malignant B cells (B-CLL). r Cumulative data of the concentrations of soluble CD26 (ng/ml) in the plasma of HC and CLL. s Cumulative data of the frequency of CD26+, and (t) CD26low, CD26high CD8+ T cell in the peripheral blood versus bone marrow of CLL. u Cumulative data of CD26 mRNA expression in CD8+ T cells of HCs vs. CLL (n=15). Error bars represent the median with an interquartile range. Each dot represents an individua [file 40164_2023_375_MOESM1_ESM.pdf]
