# Supplementary material for: Depletion of polyfunctional CD26highCD8+ T cells repertoire in chronic lymphocytic leukemia
Source: Exp Hematol Oncol. 2023 Jan 27;12:13. doi: 10.1186/s40164-023-00375-5 (PMC9881277; doi:10.1186/s40164-023-00375-5)
Supplement: Supplementary file 2 — Additional file 2: Table S1. Demographic and clinical information. [file 40164_2023_375_MOESM2_ESM.pdf]

Additional file 2: Table S1. Demographic and clinical information

Patient ID	Age/ year	Sex	CLL Stage	IGHV mutation	FISH analysis	Treatment status	Treatment Course
1	70s	He	II	Unmutated	Trisomy 12	N	-
2	50s	She	I	Not done	Not done	N	-
3	50s	He	0	Unmutated	Del 13q	N	-
4	60s	He	II	Not done	Del 13q	T	Chlorambucil+Obinutuzumab
5	70s	He	0	Not done	Del 13q	T	FCR 2 cycles
6	60s	She	0	Not done	Not done	N	-
7	80s	She	0	Not done	Not done	N	-
8	80s	He	0	Not done	Not done	N	-
9	70s	She	0	Not done	Not done	N	-
10	60s	He	I	Not done	Not done	N	-
11	80s	He	II	Not done	Not done	N	-
12	60s	He	I	Not done	Del 17p<br>Del 11q	T	FCR, RCVP, Allogenic stem cell transplant, Ibrutinib, Venetoclax, Rituximab
13	70s	He	0	Not done	Not done	N	-
14	80s	She	I	Not done	Not done	N	-
15	60s	He	III	Mutated	Del 13q	N	-
16	60s	He	II	Unmutated	Del 13q	N	-
17	70s	He	II	Unmutated	Normal	N	-
18	80s	He	III	Not done	Not done	T	Bendamustine+ Rituximab
19	70s	She	IV	Not done	Not done	T	Bendamustine+ Rituximab<br>Ibrutinib
20	70s	She	I	Not done	Not done	N	-
21	70s	She	0	Not done	Del 13q	T	Bendamustine+ Rituximab
22	60s	He	I	Not done	Del 17p<br>Del 11q	T	Ibrutinib+ Rituximab
23	60s	He	IV	Unmutated	Del 11q<br>Del 13q	T	FCR 2 cycles, Ibrutinib
24	50s	He	II	Not done	Not done	N	-
25	50s	He	II	Not done	Normal	N	_
26	70s	He	II	Not done	Normal	T	Bendamustine+ Rituximab<br>CHOP
27	90s	She	I	Not done	Not done	T	Chlorambucil+Obinutuzuma<br>Ibrutinib
28	60s	He	II	Not done	Not done	N	-
29	70s	He	IV	Not done	Del 13q<br>Trisomy 12	T	Chlorambucil+Obinutuzumab
30	60s	He	II	Not done	Del 13q	T	Ibrutinib+Venetoclax
31	60s	She	0	Not done	Not done	N	-
32	50s	She	0	Not done	Not done	N	-
33	70s	She	I	Not done	Del 13q	N	-
34	70s	She	I	Not done	Del 13q	T	Chlorambucil+Obinutuzumab
35	70s	He	III	Unmutated	Trisomy 12	N	-
36	50s	She	III	Mutated	Del 13q<br>Del 11q	N	-
37	70s	He	IV	Not done	Normal	N	-
38	40s	She	II	Mutated	Del 13q	N	-
39	60s	She	II	Indeterminate	Trisomy 12	N	-
40	70s	He	II	Unmutated	Trisomy 12	N	-
41	70s	He	I	Unmutated	Del 13q<br>Trisomy 12	N	-

NO#	Age	Sex	Stage	IGHV mutation	FISH analysis	Treatment	Treatment Course
42	70s	He	I	Not done	Del 11q	T	Ibrutinib+Obnituzumab<br>Venetoclax+Rituximab
43	50s	He	IV	Not done	Del 13q<br>Tp53 loss<br>(22%)	T	FCR+Lenalidomide<br>Ibrutinib<br>Venetoclax+Rituximab
44	70s	He	0	Not done	Not done	N	-
45	60s	He	0	Not done	Del 13q	N	-
46	70s	He	I	Indeterminate	Trisomy 12	N	-
47	30s	She	I	Unmutated	Del 17p	T	Ibrutinib
48	70s	He	I	Not done	Del 17p	T	FCR
49	50s	He	I	Unmutated	Trisomy 12	T	FCR<br>Venetoclax+Rituximab
50	50s	She	I	Mutated	Normal	N	-
51	70s	He	0	Mutated	Del 13q	N	-
52	60s	He	II	Mutated	Not done	N	-
53	60s	She	I	Not done	Not done	N	-
54	40s	He	I	Not done	Not done	N	-
55	80s	She	I	Not done	Not done	N	-

Treatment naïve (N), Treated (T), CLL-stage (Based on Rai staging system for CLL). The information was obtained at the time of T cell repertoire analysis.

Summary of Supplementary Table 1.

Characteristics	Values (Numbers)
Patients	
Total patients	55
Blood samples	48
Bone marrow samples	12
Age (yrs)	
Median	71
Range	38-91
Sex	
Female	22
Male	33
Clinical stage (Rai)	
Low (0)	13
Intermediate (I/II)	33
High (III/IV)	9
IGHV mutation	
Unmutated	10
Mutated	6
Not available/Indeterminate	37/2
FISH analysis	
Trisomy 12	7
Del 11q	5
Del 13q	15
Del 17p	4
TP53 loss+ Del 13q	1
Trisomy12+ Del 13q	1
Normal	4
Not available	18
Treatment	
